# Plasma-Catalytic CO_2_ Hydrogenation over a Pd/ZnO Catalyst: *In Situ* Probing of Gas-Phase and Surface Reactions

**DOI:** 10.1021/jacsau.2c00028

**Published:** 2022-05-31

**Authors:** Yuhai Sun, Junliang Wu, Yaolin Wang, Jingjing Li, Ni Wang, Jonathan Harding, Shengpeng Mo, Limin Chen, Peirong Chen, Mingli Fu, Daiqi Ye, Jun Huang, Xin Tu

**Affiliations:** † Guangdong Provincial Key Laboratory of Atmospheric Environment and Pollution Control, School of Environment and Energy, South China University of Technology, Guangzhou 510006, China; ‡ School of Environmental Science and Engineering, Zhejiang Gongshang University, Hangzhou 310018, China; § Department of Electrical Engineering and Electronics, University of Liverpool, Liverpool L69 3GJ, U.K.; ∥ Laboratory for Catalysis Engineering, School of Chemical and Biomolecular Engineering, Sydney Nano Institute, The University of Sydney, Sydney, NSW 2006, Australia; ⊥ National Engineering Laboratory for VOCs Pollution Control Technology and Equipment, South China University of Technology, Guangzhou 510006, China; # International Science and Technology Cooperation Platform for Low-Carbon Recycling of Waste and Green Development, Zhejiang Gongshang University, Hangzhou 310012, China

**Keywords:** plasma catalysis, CO_2_ hydrogenation, *in situ* FTIR, surface reactions, reaction pathways

## Abstract

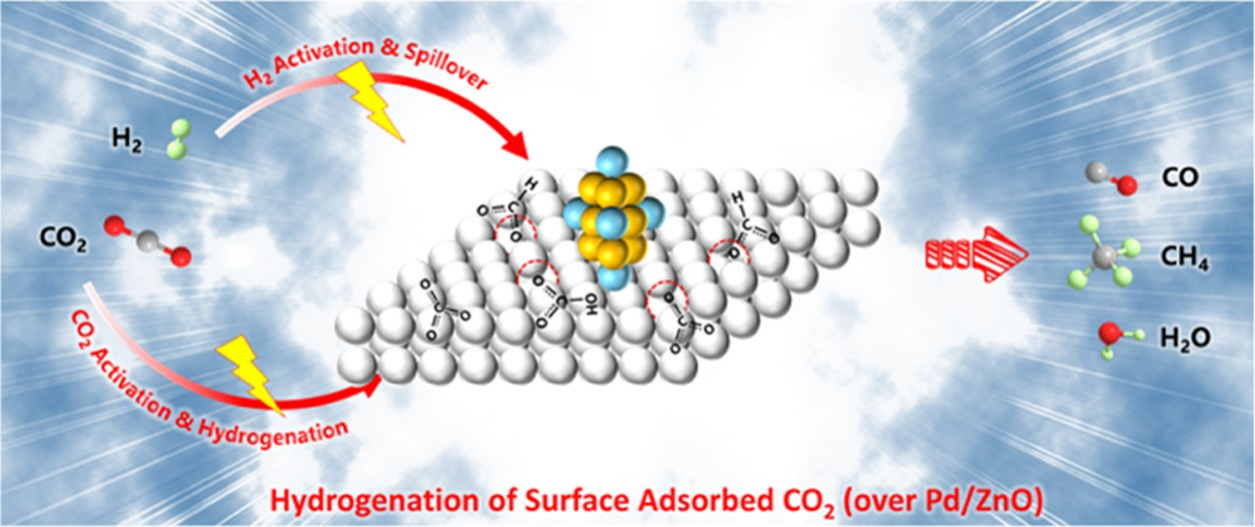

Plasma-catalytic CO_2_ hydrogenation is a complex chemical process combining plasma-assisted gas-phase and surface reactions. Herein, we investigated CO_2_ hydrogenation over Pd/ZnO and ZnO in a tubular dielectric barrier discharge (DBD) reactor at ambient pressure. Compared to the CO_2_ hydrogenation using Plasma Only or Plasma + ZnO, placing Pd/ZnO in the DBD almost doubled the conversion of CO_2_ (36.7%) and CO yield (35.5%). The reaction pathways in the plasma-enhanced catalytic hydrogenation of CO_2_ were investigated by *in situ* Fourier transform infrared (FTIR) spectroscopy using a novel integrated *in situ* DBD/FTIR gas cell reactor, combined with online mass spectrometry (MS) analysis, kinetic analysis, and emission spectroscopic measurements. In plasma CO_2_ hydrogenation over Pd/ZnO, the hydrogenation of adsorbed surface CO_2_ on Pd/ZnO is the dominant reaction route for the enhanced CO_2_ conversion, which can be ascribed to the generation of a ZnO*_x_* overlay as a result of the strong metal–support interactions (SMSI) at the Pd–ZnO interface and the presence of abundant H species at the surface of Pd/ZnO; however, this important surface reaction can be limited in the Plasma + ZnO system due to a lack of active H species present on the ZnO surface and the absence of the SMSI. Instead, CO_2_ splitting to CO, both in the plasma gas phase and on the surface of ZnO, is believed to make an important contribution to the conversion of CO_2_ in the Plasma + ZnO system.

## Introduction

The continuous consumption of fossil fuels has led to the rapid growth of CO_2_ concentrations in the atmosphere, significantly contributing to climate change and global warming. CO_2_ conversion and utilization is considered an important strategy to reduce CO_2_ emissions while producing valuable fuels and chemicals for energy storage.^1−3^ However, CO_2_ is a very stable chemical, and thus, the conversion of CO_2_ often requires high temperature and/or high pressure with the presence of a catalyst. Efficient, cost-effective, and selective reduction of CO_2_ into synthetic fuels and chemical building blocks continues to be one of the greatest challenges in the 21st Century. Significant efforts have been devoted to exploring and investigating different catalytic routes for CO_2_ valorization, such as CO_2_ hydrogenation, CO_2_ decomposition, and dry reforming of methane (DRM) with CO_2_.^4−8^ Conversion of CO_2_ with H_2_ to CO, also called the reverse water gas shift (RWGS) reaction, has received increasing interest recently, especially in conjunction with the Fischer–Tropsch process in the interest of producing hydrocarbon fuels.^9−12^ However, RWGS is an energy-intensive process as this reaction is endothermic and thus is thermodynamically favorable only at higher temperatures.

Nonthermal plasma (NTP) is an emerging technology for CO_2_ valorization under mild conditions. Energetic electrons generated by NTP can react with reactants (*e.g.*, CO_2_) or background gases and generate a cascade of active and energetic species such as ions, free radicals, excited molecules, and atoms, which might not exist in thermal or catalytic processes.^13−18^ The unique nonequilibrium character of NTP enables the progression of thermodynamically unfavorable reactions (*e.g.*, RWGS) in ambient conditions. In addition, NTP processes are instantaneous, allowing them to be switched on as needed, providing tremendous flexibility for integration with renewable energy sources such as wind and solar power, especially with the use of intermittent renewables for decentralized chemical energy storage. In addition, coupling NTP with catalysis (plasma catalysis) also offers a notable prospect of generating a synergistic effect arising from physicochemical interactions between the NTP and the catalyst, offering an effective way for the selective production of chemicals and fuels from a range of carbon-containing compounds such as CO_2_ with enhanced conversion and energy efficiency.^19−21^ For example, Zeng et al.^22^ reported a low-temperature (160 °C) synergy resulting from the coupling of a dielectric barrier discharge (DBD) NTP with promoted Ni catalysts in the plasma-enhanced catalytic DRM reaction. Combining the DBD with Ni–K/Al_2_O_3_ demonstrated the highest reaction performance, with superior conversions of CH_4_ and CO_2_ and enhanced yields of syngas (H_2_ and CO) and C_2_–C_4_ alkanes compared to that of the sum of the Plasma Only and Catalysis Only processes individually. A typical plasma-catalysis synergy was also found in the plasma-enhanced hydrogenation of CO_2_ to methanol using a Cu/γ-Al_2_O_3_ catalyst under ambient conditions.^23^

Recently, Pd/ZnO was shown to have a high activity for catalytic CO_2_ hydrogenation. Pd is effective for the activation of H_2_, generating sufficient active H species for CO_2_ hydrogenation.^24^ In addition, the strong metal–support interaction (SMSI) between Pd and ZnO can produce partially reduced ZnO (ZnO*_x_*), with the formation of abundant surface oxygen defects at the Pd–ZnO interface, which has demonstrated impressive capability for CO_2_ activation.^25,26^ Moreover, previous works confirmed that the formation of ZnO*_x_* can effectively enhance the activation of CO_2_ and spillover of H_2_ for surface CO_2_ hydrogenation.^27,28^ Despite this, the usage of Pd/ZnO in the field of plasma-catalytic CO_2_ conversion is very limited. Considering the relatively high activity of Pd/ZnO at low temperatures and pressures, it could be a very promising candidate for plasma-catalytic CO_2_ hydrogenation under mild conditions.

Plasma-catalytic chemical reactions (*e.g.*, CO_2_ hydrogenation) are a complex chemical process, with a combination of gas-phase plasma reactions and plasma-assisted surface reactions.^23^ In a typical plasma-catalytic RWGS reaction, the reactants (*i.e.*, CO_2_ and H_2_) excited by the plasma in the gas phase can be transformed into different types of reactive species including radicals, ions, and excited atoms and molecules such as CO_2_^+^, O^–^, O_2_^–^, H, O, CO, excited CO_2_, H_2_, *etc*.^29^ Along with the direct adsorption of CO_2_ and H_2_ onto the catalyst surfaces,^30^ some reactive species (*e.g.*, CO, H, excited CO_2_) generated in the plasma may be adsorbed onto the catalyst, creating extra reaction routes for CO_2_ conversion, which might not exist in thermal catalysis.^13,18^ Clearly, the gas-phase plasma reactions and surface reactions both have an impact on CO_2_ conversion. However, the exact reaction pathways in plasma-assisted catalytic CO_2_ hydrogenation (*e.g.*, RWGS) have not been fully explored and are still not clear; particularly, the plasma-assisted reactions on the surface of the catalyst, such as the formation of intermediates on the catalyst, are largely unknown.

*In situ* Fourier transform infrared spectroscopy (FTIR) is a powerful tool for probing surface reactions and has been widely used in thermal catalysis. However, the use of *in situ* FTIR to investigate plasma-induced surface reactions in the plasma-catalytic CO_2_ conversion is limited and remains a significant challenge due to the complexity present in the design of an integrated reactor coupling FTIR (*e.g.*, gas cell) with a plasma reactor.^15,20,31−34^ Combining *in situ* FTIR with advanced online spectroscopic analyses (*e.g.*, optical emission spectroscopy (OES), online mass spectrometry (MS), and plasma-assisted temperature-programmed adsorption and desorption) to elucidate the reaction mechanism in the hybrid plasma-enhanced catalytic reactions has not been investigated and would offer a promising way to obtain new insights into the plasma-induced surface reactions as well as the gas-phase plasma reactions.

In this work, the influence of ZnO and Pd/ZnO on the plasma-enhanced catalytic hydrogenation of CO_2_ to CO was explored using a typical tabular DBD reactor. Comprehensive catalyst characterization was carried out including unique plasma-assisted temperature-programmed desorption (H_2_-TPD and CO_2_-TPD). A novel integrated reactor combining a DBD with an FTIR gas cell was designed for *in situ* characterization of plasma-assisted surface reactions. *In situ* FTIR combined with online MS and OES analysis was used to investigate the effect of ZnO and Pd/ZnO on the plasma-assisted gas-phase and surface reactions, particularly regarding the generation of any intermediates on the catalyst surfaces in the plasma-catalytic CO_2_ hydrogenation. Coupling these results with kinetic analysis, alternate reaction pathways for the plasma-enhanced catalytic CO_2_ hydrogenation were proposed and discussed.

## Results and Discussion

### Plasma-Catalytic CO_2_ Hydrogenation

**Figure 1:**
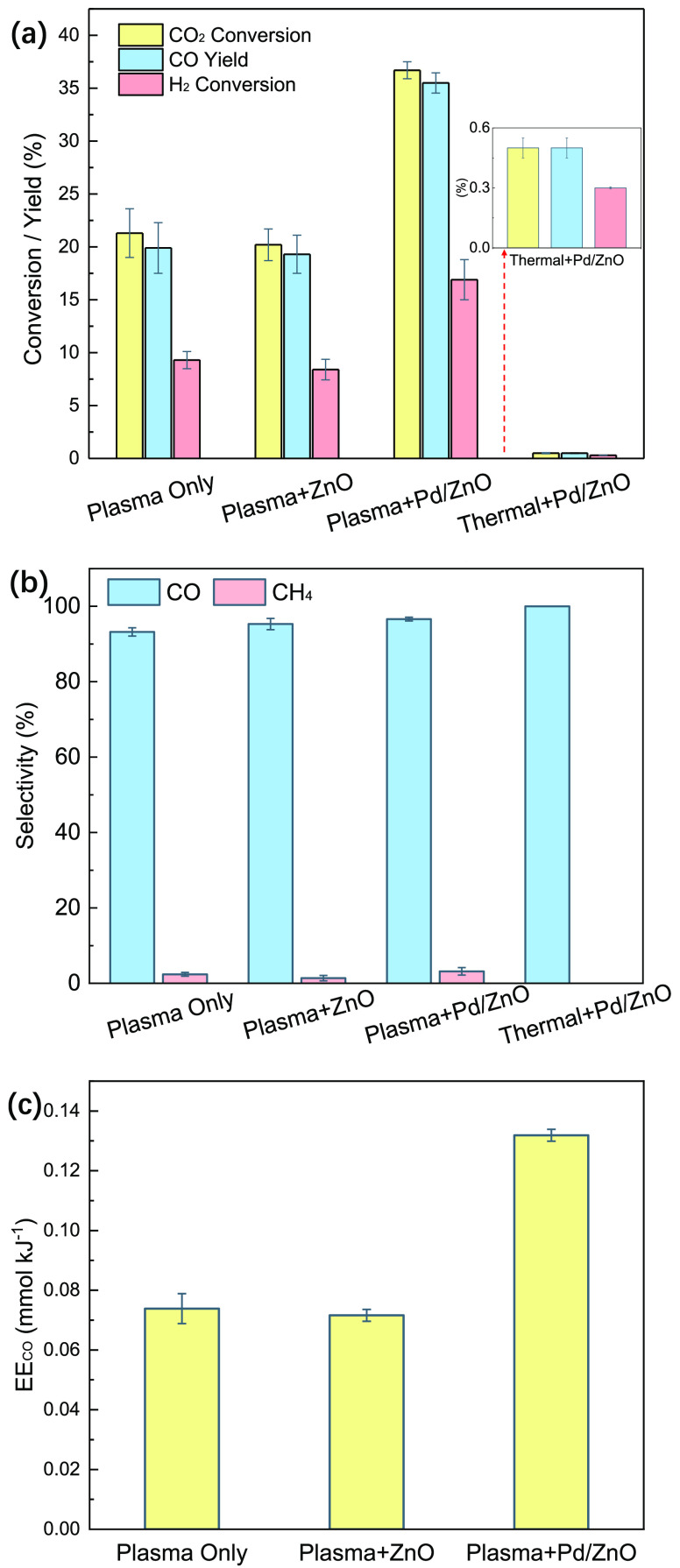
Performance of CO_2_ hydrogenation in different plasma systems (gas hourly space velocity = 2200 h^–1^, total flow rate = 40 mL min^–1^, H_2_/CO_2_ = 3:1; reaction temperature = 200 °C for thermal catalytic CO_2_ hydrogenation; discharge power = 20 W for plasma reactions). Note the error bar for the CO selectivity using Thermal + Pd/ZnO was not provided as it was always 100% in the repeated measurements.

Plasma CO_2_ hydrogenation was carried out with and without packing in a DBD reactor (see details in the Experimental Section and Figure S1). The conversion of CO_2_ and H_2_ was 21.3 and 9.3%, respectively, in the plasma reaction with no packing (Figure 1a). However, placing ZnO in the DBD did not enhance the conversion of CO_2_ (20.2%) and H_2_ (8.4%). This finding could be attributed to reduced gas-phase reactions due to a packed-bed effect in the Plasma + ZnO system and limited surface hydrogenation reactions due to the weak catalytic activity of ZnO. In contrast, the combination of DBD with 2 wt % Pd/ZnO notably improved the conversion of CO_2_ and H_2_ to 36.7 and 16.9%, respectively. This enhancement could be partially attributed to the formation of a ZnO*_x_* overlayer with the presence of richer oxygen vacancies on the Pd/ZnO catalyst caused by the SMSI between ZnO and Pd, which is evidenced by high-resolution transmission electron microscopy (HRTEM) and X-ray photoelectron spectroscopy (XPS) analyses (Figures S2 and S3 and Table S1). The presence of the ZnO*_x_* overlayer can effectively activate both H_2_ and CO_2_ for the surface CO_2_ hydrogenation. Despite this, the conversion of both H_2_ and CO_2_ was less than 1% in the thermal catalytic CO_2_ hydrogenation at 200 °C (Figures 1a and S4). Note that we found that an increase of the Pd loading from 2 to 5 wt % provided only a slight increase of the CO_2_ conversion to 40.2% but decreased the CO selectivity to ∼85% in the CO_2_ hydrogenation using plasma catalysis (Figure S5). A similar finding was noted by Wang et al. when looking at thermal catalytic CO_2_ reduction.^35^ Considering the cost of Pd and the activities of Pd/ZnO with different Pd loadings, we chose 2 wt % Pd loading in this study.

In comparison to the Plasma Only system, the incorporation of plasma with ZnO or Pd/ZnO showed a similar CO selectivity (93.2–96.6%) (Figure 1b). This result is consistent with those published in the previous literature where CO is the major gas product in plasma CO_2_ hydrogenation (i.e., RWGS reaction) using conventional coaxial DBD reactors.^18^ The presence of packing materials (including catalysts) and their effect on the CO selectivity in the plasma RWGS is limited as the CO selectivity is typically higher than 90% even without using packing. The presence of ZnO or Pd/ZnO in the plasma discharge also had only a minor effect on the selectivity of CH_4_ (1.4–3.2%) in comparison to using Plasma Only. Note that no oxygenates were detected in this reaction. Interestingly, we found that the presence of Pd/ZnO almost doubled the CO yield (35.5%) when comparing against the same reaction with Plasma Only (19.9%) and Plasma + ZnO (19.3%) due to significantly enhancing the CO_2_ conversion while maintaining a similar CO selectivity. Due to this, the energy efficiency for CO production also received a boost when using Pd/ZnO. In this study, the energy efficiency for CO production (up to 0.13 mmol kJ^–1^, Figure 1c) was greater than those published in previous works under similar conditions.^36,37^

Figure 1 shows a plasma-catalytic synergy for both CO_2_ conversion and CO yield, as well as the significance of Pd in the plasma hydrogenation of CO_2_ over the Pd/ZnO catalyst. The plasma-catalytic synergy (CO_2_ conversion and CO yield) over the 2 wt % Pd/ZnO catalyst was also confirmed when changing the gas flow rate (40–120 mL/min) and discharge power (10–20 W), as shown in Figure S6. The time-on-stream experiments showed that the CO_2_ conversion was very stable over the 6 h reaction regardless of the use of a catalyst or support (Figure S7).

### Effect of Catalysts on Discharge Characteristics

Figure S8 shows the influence of the packing material on the electrical signals of the discharge at a constant power of 20 W. The total current of the DBD showed a typical quasi-sinusoid signal with a great number of superimposed current pulses. The DBD without packing was dominated by filamentary discharges. Compared to the discharge without packing, the combination of DBD with either ZnO or Pd/ZnO decreased the current amplitude, suggesting that the presence of filamentary discharges passing through the gas gap was weakened due to a packed-bed effect and reduced void space. Comparing this to the DBD without packing, the current pulses appeared denser in the DBD incorporated with Pd/ZnO due to the formation of more filamentary discharges propagating along the surface of Pd/ZnO. A comparable phenomenon was also reported in previous studies.^38−40^ The enhanced surface reactions in the Plasma + Pd/ZnO system could contribute to the higher CO_2_ conversion and CO yield in the plasma-catalytic CO_2_ hydrogenation when compared to the plasma reaction without packing. Notably, placing ZnO or Pd/ZnO into the DBD showed similar electrical characteristics (current, applied voltage, and Lissajous figure, see Figure S8), which could be a result of the low Pd loading (2 wt %) on ZnO. This finding also suggests that the different reaction performances (*e.g.*, CO_2_ conversion) using ZnO and Pd/ZnO could be mainly associated with the catalytic activities and properties of these materials rather than the discharge properties induced by these materials.

Figure S9 shows the emission spectra of the CO_2_/H_2_ DBD with and without packing. CO_2_^+^ (A^2^Σ^+^ → X^2^Π, A^2^Π → X^2^Π) and CO (b^3^Σ^+^ → a^3^Π, B^1^Σ^+^ → A^1^Π) molecular bands were observed in the CO_2_/H_2_ DBD regardless of whether a packing was used.^23^ A hydrogen atomic line (H_α_) at 656.3 nm was found in the spectrum of the CO_2_/H_2_ DBD without packing. However, H_α_ was not detected in the OES of the discharge coupled with either ZnO or Pd/ZnO, which might be attributed to the weakened filamentary discharges passing through the gas gap.

### Influence of Catalysts on the Adsorption of H_2_ and CO_2_

**Figure 2:**
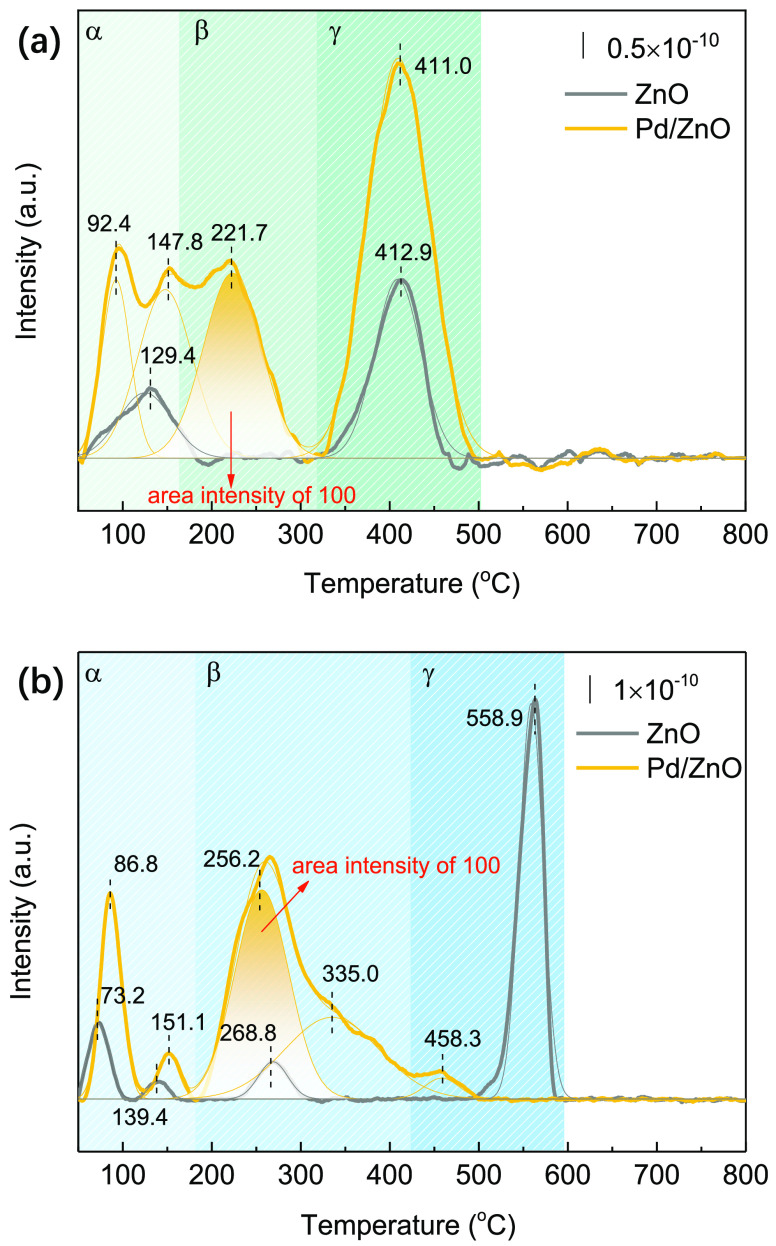
Adsorption and activation of H_2_ and CO_2_ on the surface of ZnO and Pd/ZnO using plasma-coupled TPD characterization: (a) H_2_-TPD and (b) CO_2_-TPD.

The adsorption and activation of H_2_ and CO_2_ on different surfaces (ZnO and Pd/ZnO) were investigated using the plasma-coupled H_2_-TPD and CO_2_-TPD experiments, respectively (see details in the Experimental Section). The same DBD reactor used for CO_2_ hydrogenation was integrated with the conventional TPD processes. Figure 2a shows the adsorption of H_2_ on the surface of ZnO and Pd/ZnO. The H_2_-TPD profile for both ZnO and Pd/ZnO spanned a wide temperature range (50–500 °C) with three temperature zones (α, β, and γ). The peaks below 150 °C represent the weak desorption of H_2_ over Pd and ZnO. The desorption peak between 300 and 500 °C is associated with the irreversible desorption of H_2_ from the surface of ZnO and Pd/ZnO.^41^ Compared with ZnO, Pd/ZnO exhibited a new desorption peak at 221.7 °C, suggesting the presence of H_2_ spillover from the highly dispersed Pd nanoparticles (NPs) to ZnO*_x_*, which is critical for hydrogenating the adsorbed CO_2_ on the Pd/ZnO surface.^27,42,43^ Additionally, loading Pd to ZnO greatly enhanced the total amount of H_2_ adsorption, from 109.0 for ZnO to 461.2 for Pd/ZnO (Table S2). Figure 2b shows the adsorption states of CO_2_ on the basic sites of ZnO. The peaks below 180 °C are associated with the presence of physically/weakly adsorbed CO_2_ on ZnO, while the peak at ∼250 °C is related to the desorption of bidentate carbonates on the medium basic sites of ZnO. The peak above 450 °C represents the decomposition of monodentate carbonates formed by strong adsorption of CO_2_ on the strong basic sites of ZnO.^44−46^ Compared to ZnO, loading Pd onto ZnO increased the total amount of CO_2_ adsorption to 220.1 (Table S3). In addition, the formation of ZnO*_x_* on account of the SMSI between Pd and ZnO modified the basicity of the Pd/ZnO catalyst and thus increased the desorption of CO_2_ moderately bound to the ZnO surface from 9.7 (for ZnO) to 168.3, which is favorable for CO_2_ conversion.^47,48^ These results suggest that Pd/ZnO is much more favorable for the activation of both H_2_ and CO_2_ compared to ZnO.

### Reaction Mechanism

#### Online MS Analysis of Surface Reactions

**Figure 3:**
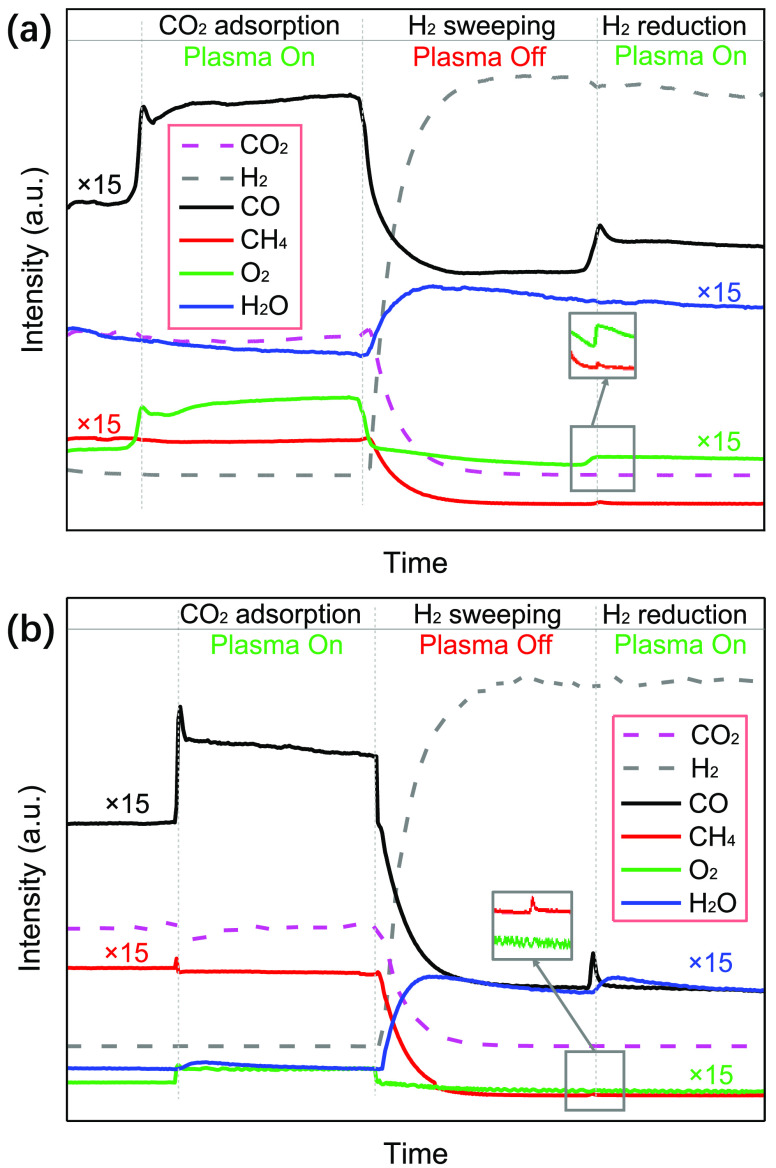
Online MS analysis of plasma-catalytic CO_2_ hydrogenation over (a) ZnO-packed and (b) Pd/ZnO-packed DBD systems.

Plasma-catalytic CO_2_ hydrogenation with ZnO and Pd/ZnO was investigated using online MS analysis through the following three steps (see details in the Experimental Section): (1) adsorption of CO_2_ with plasma on;^32^ (2) H_2_ sweeping with plasma off; and (3) conversion of surface-adsorbed CO_2_ with H_2_ (plasma on). In the CO_2_ adsorption process over ZnO and Pd/ZnO (plasma on), the appeared CO and O_2_ signals can be ascribed to CO_2_ splitting to CO and O_2_ during the plasma process.^28^ In the H_2_ plasma hydrogenation of CO_2_ adsorbed onto the ZnO surface, CO and O_2_ were detected instead of CO and H_2_O, as presented in Figure 3a. This finding reveals that the dissociation of the adsorbed CO_2_ to CO and O_2_ dominated on the surfaces of ZnO, while CO_2_ hydrogenation to CO and H_2_O has been limited due to the absence of surface hydrogen species on ZnO. However, both H_2_O and CO were detected in the H_2_ plasma hydrogenation of CO_2_ on Pd/ZnO, while O_2_ disappeared (Figure 3b), indicating that hydrogenating the adsorbed surface CO_2_ on Pd/ZnO plays a dominant role in the formation of CO as Pd has an excellent capability to activate H_2_ and provides surface reactive hydrogen species for CO_2_ hydrogenation, which has also been confirmed by the H_2_-TPD of Pd/ZnO.

#### *In Situ* FTIR Analysis of Surface Reactions

**Figure 4:**
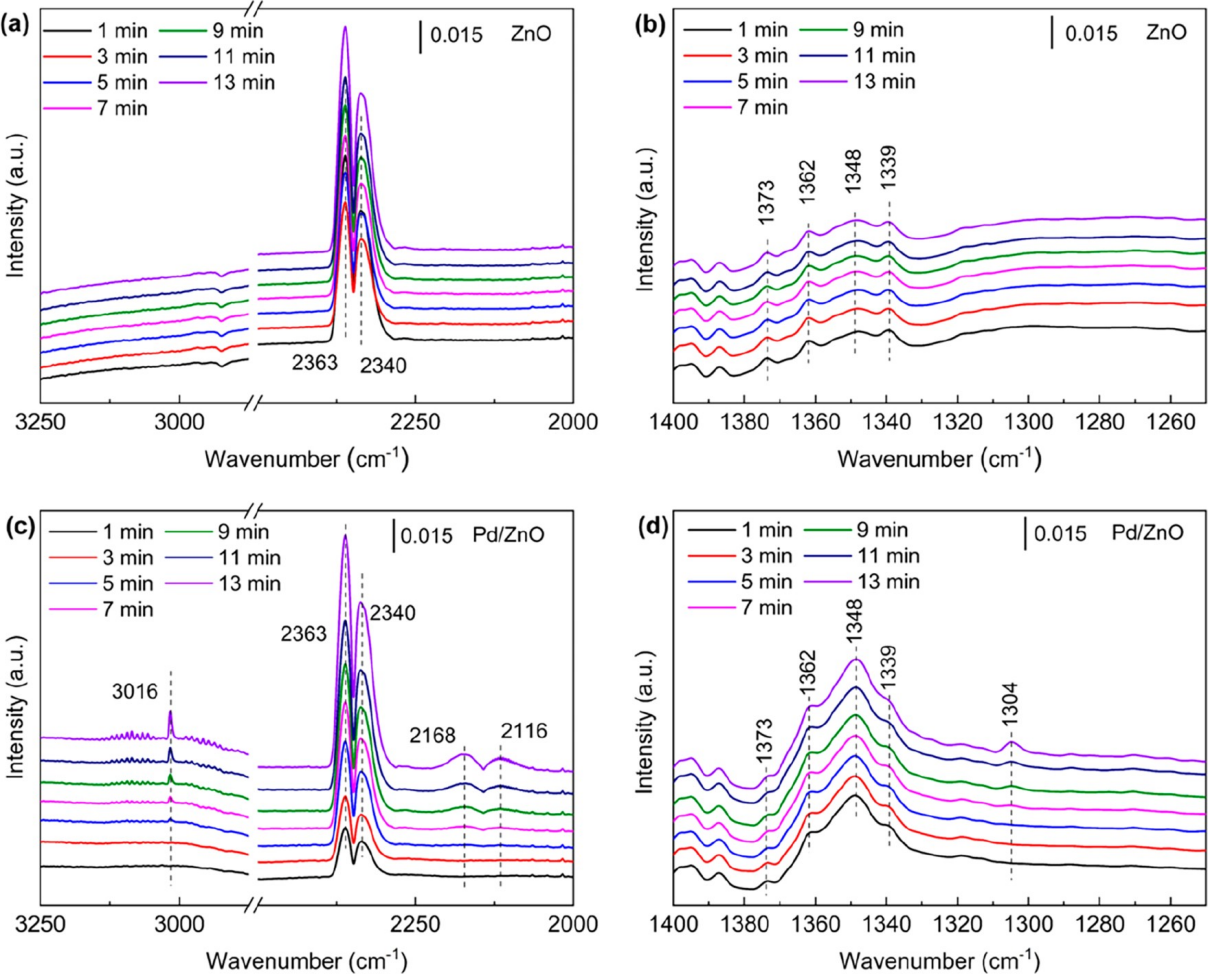
*In situ* FTIR analysis of the plasma-catalytic H_2_ hydrogenation of surface-adsorbed CO_2_ over (a, b) ZnO and (c, d) Pd/ZnO.

*In situ* FTIR was used to develop an understanding regarding the formation of surface species on the surface of ZnO and Pd/ZnO in the plasma-catalytic hydrogenation of CO_2_ using a custom-designed *in situ* DBD/FTIR reactor (see details in the Experimental Section and Figures S10 and S11). In Figure 4a–c, the intensified signal of gas-phase CO_2_ (2363 and 2340 cm^–1^)^49,50^ in the closed DBD system can be assigned to the desorption of weakly adsorbed CO_2_ from the surface of ZnO and Pd/ZnO under plasma conditions. Although the online MS analysis (Figure 3a) shows that the dissociation of adsorbed CO_2_ to CO was dominant on the surface of ZnO, no obvious signals of gas-phase CO (2120 and 2174 cm^–1^)^49^ were detected in Figure 4a. This finding can be ascribed to the limited CO_2_ dissociation in the *in situ* FTIR characterization. Unlike plasma CO_2_ hydrogenation with ZnO, the signals of gas-phase CO and CH_4_ (at 3016 cm^–1^)^51^ can be detected in the Plasma + Pd/ZnO system (Figure 4c), being accompanied by much more intensified signals of surface-adsorbed species: the peaks at 1339 and 1304 cm^–1^ are assigned to the adsorbed carbonate species,^34,51−53^ while the bands found at 1373, 1362, and 1348 cm^–1^ are ascribed to the formation of symmetric and antisymmetric OCO vibrations of formate-like species (Figure 4b,d), revealing the formation of both HOCO and HCOO surface intermediates in CO_2_ hydrogenation.^49−51^ Note that more formate-like surface species were formed on Pd/ZnO than on ZnO (Figure S12). This finding may be ascribed to the presence of ZnO*_x_* on Pd/ZnO induced by the SMSI between ZnO and Pd (Figure S3). The formation of rich oxygen vacancies on ZnO*_x_* at the Pd–ZnO interface enhanced CO_2_ adsorption, thus forming surface carbonate species (Figure S2b and Table S1).^32^ Moreover, the H_2_-TPD results confirm that the presence of ZnO*_x_* can also enhance the H_2_ spillover (Figure 2a) and produce more active H species for the hydrogenation of carbonate to formate species, thus boosting the conversion of adsorbed CO_2_.^28^ In contrast, ZnO has a low capability for converting surface-adsorbed CO_2_ (CO_2,ads_) given the absence of the SMSI. These results indicate that the surface hydrogenation reactions contribute significantly to the plasma-catalytic CO_2_ hydrogenation over Pd/ZnO, which can also be evidenced by the online MS analysis (Figure 3b). By contrast, the reaction of H_2_ with the surface-adsorbed CO_2_ is limited in the Plasma + ZnO system, which can be confirmed by the formation of more formate and carbonate species on the surface of Pd/ZnO via surface hydrogenation reactions compared to ZnO.

### Carbon Deposition

**Figure 5:**
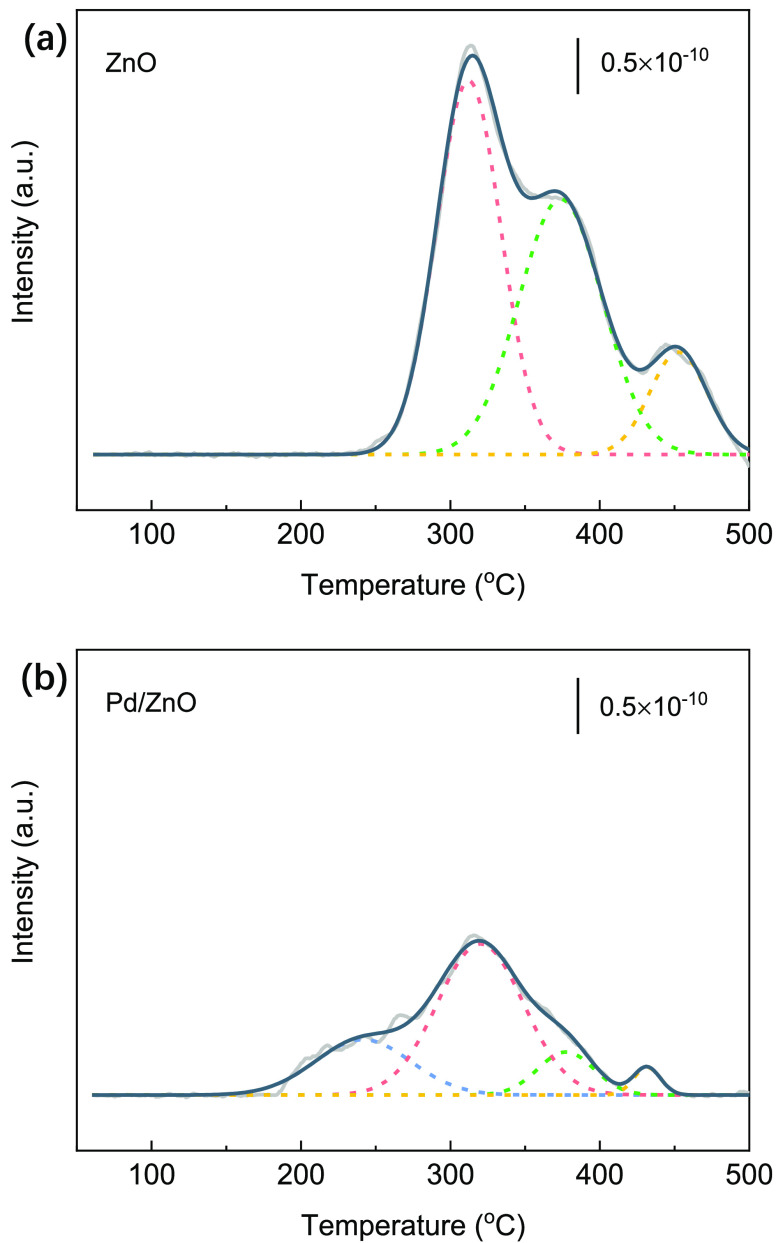
O_2_-TPO characterization of the spent (a) ZnO and (b) Pd/ZnO (after 6 h reaction).

The carbon balance in plasma CO_2_ hydrogenation was 94.0–99.0% (Figure S13), suggesting that carbon deposition was limited in this process. Catalyst characterization also confirms that the properties (*e.g.*, pore size, crystal structure, Pd chemical state, and morphology) of the Pd/ZnO catalyst were almost unchanged before and after 6 h plasma reaction (Figures S14–S19), which agrees with the results of the catalyst stability test (Figure S7). Figure 5 shows the O_2_-TPO analysis of the spent ZnO and Pd/ZnO after running plasma-catalytic CO_2_ hydrogenation for 6 h. The peak at *ca.* 250 °C can be associated with the removal of easily oxidizable carbonaceous species such as coke-containing hydrogen species and/or surface carbon.^44^ The peaks between 300 and 500 °C are associated with the combustion of amorphous carbon and/or graphitic carbon. As shown in Figure 5, more carbon was formed on ZnO than on Pd/ZnO. In addition, the carbon deposited on the spent ZnO would be more difficult to be gasified, requiring a higher burning temperature compared to Pd/ZnO. The online MS analysis combined with *in situ* FTIR confirms that the production of CO on the ZnO surface mainly proceeds via the dissociation of adsorbed CO_2_ in the Plasma + ZnO system, and thus has the potential to produce more carbon on the surface. In contrast, CO generation on the Pd/ZnO surface is dominated by surface hydrogenation of carbonate species, resulting in less carbon deposition.

#### Kinetic Analysis

**Figure 6:**
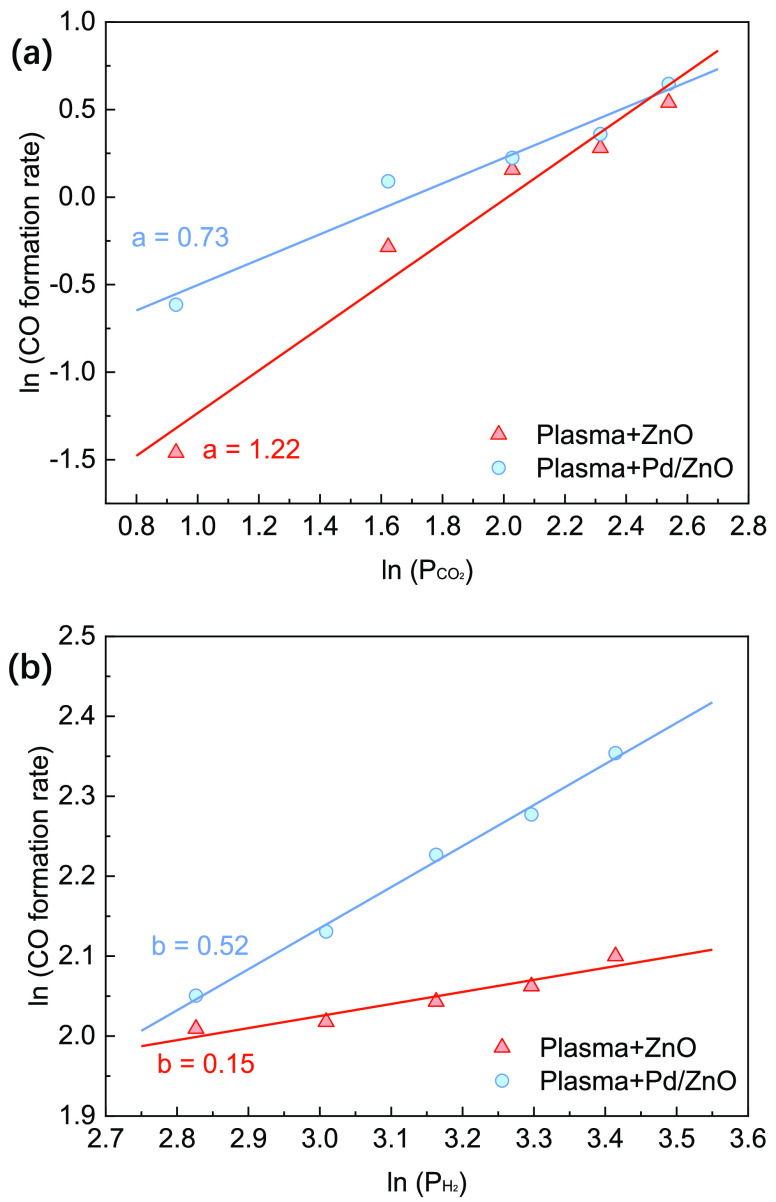
Reaction orders of (a) CO_2_ and (b) H_2_ in plasma CO_2_ hydrogenation packed with ZnO and Pd/ZnO.

In this study, we found that the active H species formed on the catalyst surface are crucial for the hydrogenation of surface-adsorbed CO_2_ for CO production. Therefore, the CO production rate was determined at different partial pressures of CO_2_ (Figure 6a) when keeping the partial pressure of H_2_ constant and vice versa (Figure 6b). Packing the discharge gap in a DBD reactor with supports or catalysts typically changes the discharge mode and properties (Figure S6). Thus, the kinetic analysis was carried out only considering the plasma CO_2_ hydrogenation in the presence of ZnO and Pd/ZnO. A similar approach was adopted for the kinetic analysis by Barboun et al.^54^Figure 6a shows the reaction order was 1.22 and 0.73 for Plasma + ZnO and Plasma + Pd/ZnO, respectively, suggesting CO_2_ has a positive effect on CO production with the packing of ZnO and Pd/ZnO. However, the reaction order of H_2_ was much lower than that of CO_2_ (Figure 6b). These findings imply that the CO_2_ concentration is more likely to influence the reaction rate for CO production in comparison to that of H_2_. In addition, the H_2_ reaction order for Pd/ZnO (0.52) was higher than that of bare ZnO (0.15), suggesting that the presence of Pd sites can change the kinetic behavior of plasma CO_2_ hydrogenation distinctly.^55^

### Reaction Pathways

**Figure 7:**
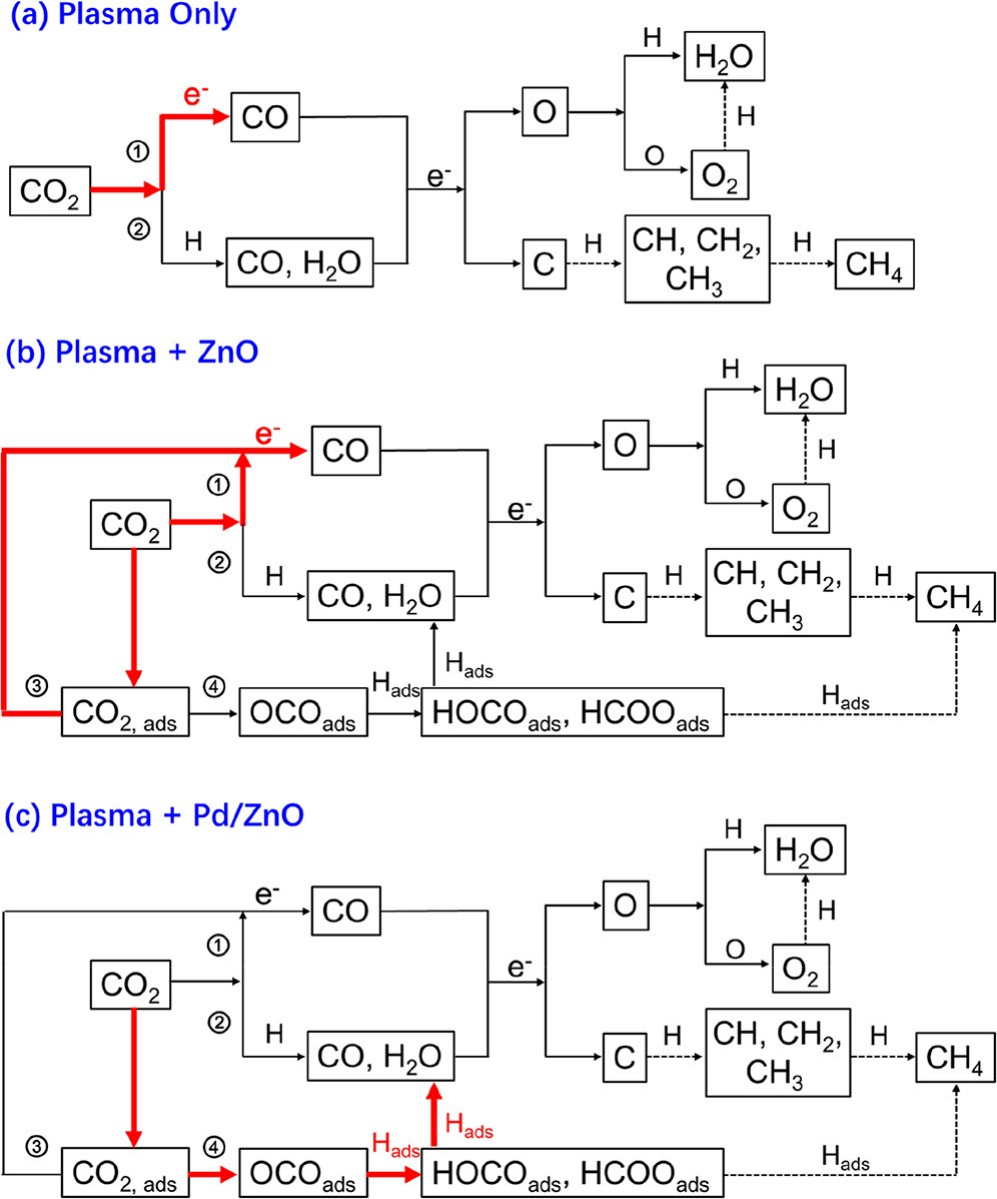
Reaction pathways for the conversion of CO_2_ in different plasma systems, (a) Plasma Only, (b) Plasma + ZnO, and (c) Plasma + Pd/ZnO (red arrow: primary reaction pathway; black arrow: secondary reaction pathway; dashed black arrow: estimated reaction pathway; ads subscript: surface-adsorbed species).

Figure 7 displays the proposed reaction pathways of plasma CO_2_ hydrogenation with and without packing.

(1) During plasma CO_2_ hydrogenation without packing (Figure 7a), electron impact CO_2_ dissociation is the main reaction for CO_2_ conversion and CO production (pathway ①), as demonstrated by a one-dimensional (1D) fluid modeling study.^56^ The modeling results also showed that electron impact dissociation of H_2_ is the major process for the activation of H_2_ to H atoms although a number of H atoms can recombine to H_2_. In this work, the presence of H atomic lines has also been confirmed in the emission spectrum of the CO_2_/H_2_ DBD without packing. In addition, CO_2_ hydrogenation also contributes to the conversion of CO_2_ in the gas phase (pathway ②). Following the dissociation of CO_2_ and H_2_, H atoms can recombine with CO to form HCO, an unstable intermediate, which can react with a H atom to produce H_2_ and CO, representing another route for CO production.^56^ Although CO can be further decomposed to carbon and O via electron impact dissociation of CO, the relative contribution of this reaction pathway in this process is very limited.^56^ Note the carbon formed in the reaction can react with H or H_2_ to generate CH or CH_2_, respectively, both of which are regarded as important precursors for generating CH_4_ via step-wise hydrogenation in the gas phase.^57^ Due to the limited contribution of the electron impact dissociation of CO to C in the gas-phase plasma reactions, the formation of CH_4_ in plasma CO_2_ hydrogenation without a methanation catalyst is very limited.
(2) In plasma CO_2_ hydrogenation over ZnO (Figure 7b), the breaking of CO_2_ to CO and O_2_ plays a dominant role in the conversion of CO_2_, in both the gas phase (pathway ①) and on the surface of ZnO (pathway ③). This can be confirmed through the online MS analysis, which reveals that CO_2_ decomposition to CO and O_2_ occurs in the Plasma + ZnO system (Figure 7b). In addition, CO_2_ hydrogenation in the gas phase also plays a part in the conversion of CO_2_ (pathway ②). The hydrogenation of adsorbed CO_2_ (pathway ④) on the ZnO surface is however limited due to the absence of the SMSI and the lack of active H species generated on the ZnO surface, as confirmed in the H_2_-TPD analysis of ZnO (Figure 2a). The presence of ZnO in the DBD slightly reduced the CO_2_ conversion when compared with the Plasma Only reaction; this could be attributed to the reduced formation of filament discharges passing through the gas gap and the limited hydrogenation of surface CO_2_ on ZnO, as evidenced by the combined electrical diagnostics and *in situ* FTIR and online MS analysis.
(3) In plasma CO_2_ hydrogenation over Pd/ZnO (Figure 7c), hydrogenating adsorbed surface CO_2_ on Pd/ZnO (pathway ④) is a dominant reaction route contributing to the enhanced CO_2_ conversion due to the presence of abundant H species (evidenced by H_2_-TPD) on the surfaces of Pd/ZnO via H_2_ activation by Pd NPs. *In situ* FTIR analysis further confirms that CO_2_ can be adsorbed onto the surfaces of Pd/ZnO to form OCO species, which can be further hydrogenated to HOCO and HCOO for the production of CO. By contrast, the decomposition of adsorbed CO_2_ to CO and O_2_ on Pd/ZnO is eliminated, which can be demonstrated through the online MS analysis with H_2_O instead of O_2_ being detected in the surface reactions.

## Conclusions

In summary, we investigated plasma-catalytic CO_2_ hydrogenation over ZnO and Pd/ZnO using a tabular DBD reactor at low temperatures. Combining plasma with Pd/ZnO significantly enhanced CO_2_ conversion and CO yield when compared to the Plasma Only reaction or Plasma + ZnO. *In situ* spectroscopy techniques including *in situ* FTIR, online MS and OES diagnostics combined with catalyst characterization, and kinetic analysis were used to understand the role of Pd/ZnO in the plasma-catalytic CO_2_ hydrogenation, particularly to develop a new understanding of the formation of intermediates on the catalyst surfaces. In the plasma-catalytic reaction using Pd/ZnO, the hydrogenation of adsorbed CO_2_ on Pd/ZnO significantly contributes to the enhanced CO_2_ conversion, which can be attributed to the formation of a ZnO*_x_* overlay as a consequence of the SMSI between ZnO and Pd, and the presence of abundant H species (confirmed by plasma-assisted H_2_-TPD analysis) on the Pd/ZnO surface due to H_2_ activation by Pd NPs. However, without Pd loading, the hydrogenation of surface-adsorbed CO_2_ on the ZnO surface was limited due to the absence of the SMSI and lack of active H species formed on the ZnO surface. The splitting of CO_2_ to CO is believed to make major contributions to the conversion of CO_2_ in both the gas phase and on the ZnO surface during the plasma-catalytic CO_2_ hydrogenation over ZnO. The designed novel integrated DBD/FTIR gas cell reactor coupled with online MS and OES diagnostics offers a promising solution to develop a greater comprehension of the reaction mechanisms and pathways for complicated plasma-catalytic chemical reactions, particularly plasma-assisted surface reactions.

## Experimental Section

### Catalyst Preparation

The Pd/ZnO catalysts (2 and 5 wt % Pd) were prepared using a coprecipitation method. To prepare 2 wt % Pd/ZnO, a mixture of Pd(NO_3_)_2_ (0.54 g, 18.01 wt % Pd, Macklin) and Zn(NO_3_)_2_·6H_2_O (14.6 g, Aladdin) was dissolved in deionized water (80 mL) and used as a precursor solution. The precursor solution and the precipitant agent, mixture of Na_2_CO_3_ (0.25 mol L^–1^) and NaOH (0.25 mol L^–1^), were then added simultaneously to a three-necked flask containing 100 mL of deionized water with vigorous stirring at 60 °C to keep the pH value of the precursor solution at 9.0–9.5. The resulting solution was then aged at 60 °C for 4 h under continuous stirring and separated by centrifugation. The obtained sample was dried in an oven at 100 °C for 10 h and then calcined in a tube furnace using dry air for 4 h (at 350 °C). Pd/ZnO (5 wt %) was synthesized using the same procedure. ZnO was prepared using a similar method but without the addition of Pd(NO_3_)_2_ for the preparation of the precursor solution. Both Pd/ZnO and ZnO samples were sieved to 40–60 mesh.

### Experimental Setup

Plasma hydrogenation of CO_2_ was conducted in a cylindrical DBD reactor, as shown in Figure S1. The detailed configuration and dimension of the reactor can be found in our previous work.^32^ The plasma reactor was connected to a high-voltage alternating current power supply (Suman, CTP-2000K). A mixture of H_2_ and CO_2_ with an H_2_/CO_2_ ratio of 3:1 was used. The catalyst (0.5 g) was packed into the entire discharge gap and was reduced by H_2_/Ar mixed gas (H_2_/Ar = 1:9) at 400 °C for 4 h before the reaction. We measured the applied voltage using a Tektronix high-voltage probe (P6015A) and the current with a Tektronix current monitor (TCP0030). The voltage on the external capacitor was sampled using a Tektronix P6139B probe. All of the electrical signals were recorded using an oscilloscope (Tektronix DPO 3034). The plasma power was determined using the typical Lissajous figure approach.

We measured the temperatures in the plasma zone using an infrared thermometer. The temperatures in the discharge zone without packing were lower than 180 °C at 20 W and 40 mL min^–1^, while the presence of Pd/ZnO or ZnO slightly increased the temperature of the catalyst bed to 180–190 °C under the same conditions. The gaseous products were analyzed using an online gas chromatograph (Shimadzu 2014C) fitted with dual detectors. OES measurements were employed to investigate the chemically active species formed in the plasma CO_2_ conversion with and without a catalyst using a spectrometer (Princeton Instruments 320PI) equipped with a focal length of 320 nm.^38^

### Catalyst Characterization

#### N_2_ Physisorption

N_2_ physisorption was performed at 77 K using an automated gas adsorption device (ASAP 2010, Micromeritics Instrument). The samples were degassed at 473 K for 2 h under vacuum before N_2_ physisorption measurements. The Brunauer–Emmett–Teller (BET) method was used to determine the specific surface area (SSA) of the samples.

#### X-ray Diffraction (XRD)

XRD was performed using a Bruker X-ray diffractometer (D8 ADVANCE) fitted with a Cu Kα radiation source with the tube voltage and current being 40 kV and 40 mA, respectively, alongside a wavelength of 0.15418 nm. The diffraction patterns were recorded using a step size of 0.02° in a 2θ range of 20–80°.

#### Electron Microscopy Analysis

The morphology and element mapping of the samples were measured on a field emission scanning electron microscope (FE-SEM), Merlin (Carl Zeiss). HRTEM images of the catalysts were recorded using an FEI Titan 60-300 cubed electron microscope. This FEI Titan 60-300 cubed electron microscope was also used to perform scanning TEM-high-angle annular dark field (STEM-HAADF) analysis.

#### XPS Analysis

XPS analysis was run using a Thermo Fisher Scientific spectrometer (ESCALAB 250Xi) using an Mg Kα radiation source with an energy of 1253.6 eV and a resolution of 0.1 eV. The C 1s peak at 284.8 eV was used to reference the binding energies.

#### Plasma-Coupled TPD Experiments

The adsorption and activation of H_2_ and CO_2_ on different surfaces (ZnO and Pd/ZnO) were investigated using plasma-coupled H_2_-TPD and CO_2_-TPD, respectively. The same DBD reactor used for plasma CO_2_ hydrogenation was integrated with the TPD process. In a typical plasma-coupled TPD analysis, the adsorption of the reactant (H_2_ or CO_2_) on the surface (Pd/ZnO or ZnO) was performed when the H_2_ (or CO_2_) DBD plasma was switched on, while the desorption process was carried out by increasing the temperature without plasma. For the plasma-coupled H_2_-TPD analysis, the calcined catalyst (0.1 g) was initially reduced by H_2_/Ar (H_2_/Ar = 1:9, total flow 30 mL min^–1^) at 400 °C for 4 h, which was proceeded by flushing Ar to 50 °C. The adsorption of H_2_ on the catalyst was then carried out in H_2_ DBD plasma for 1 h at 20 W. Next, the plasma was turned off before the catalyst was swept by flowing Ar for 2 h. Subsequently, H_2_ desorption (without plasma) began by increasing the temperature from 50 to 800 °C at a heating rate of 10 °C min^–1^ in Ar flow, and the H_2_ signal (*m*/*z* = 2) was measured using a Hiden Analytical quadrupole mass spectrometer (HPR20). The plasma-coupled CO_2_-TPD of ZnO and Pd/ZnO followed the same procedure.

#### O_2_-TPO

O_2_-TPO was carried out to characterize carbon deposited on Pd/ZnO and ZnO after operating the plasma reaction for 6 h. In a typical O_2_-TPO measurement, the spent catalyst or support (0.1 g) was heated from 60 to 500 °C at 10 °C min^–1^ in 5 vol % O_2_/He (total flow 30 mL min^–1^). An online MS (Hiden Analytical, HPR20) was used to track the evolution of the CO_2_ signal (*m*/*z* = 44) in the O_2_-TPO analysis.

### Online MS Analysis

For online MS analysis, the DBD reactor (same reactor as the CO_2_ hydrogenation reaction) was packed with the relevant catalyst or support (0.5 g), and the reactant (CO_2_ or H_2_) was diluted with Ar due to the limited inlet H_2_ concentration (up to 15 vol %) allowed for the mass spectrometer (Hiden Analytical HPR20). In addition, CO_2_ adsorption was not performed in pure CO_2_ plasma as using pure CO_2_ plasma can lead to more O_2_ (produced by CO_2_ splitting) being adsorbed on the catalyst surface, which might influence the subsequent surface hydrogenation process. In a typical experiment using the online MS analysis, when the plasma was switched on at 20 W, a mix of CO_2_ and Ar (CO_2_/Ar = 1:10) was flushed through the catalyst for adsorption, followed by switching off the plasma and sweeping with a H_2_/Ar (H_2_/Ar molar ratio of 1:10) flow to remove gas-phase CO_2_. Afterward, the hydrogenation of surface-adsorbed CO_2_ was performed in the same DBD reactor using H_2_/Ar (H_2_/Ar molar ratio of 1:10) at 20 W. Analysis of the products from H_2_/Ar plasma-assisted surface reactions was conducted using online MS (Hiden Analytical HPR20).

### *In Situ* FTIR Characterization of the Catalyst Surface under Plasma Conditions

Plasma-assisted CO_2_ conversion was monitored *in situ* using an FTIR spectrometer (Thermo NICOLET iS50) on the transmission infrared mode fitted with an HgCdTe detector with a resolution of 4 cm^–1^ using 32 scans. Figures S10 and S11 show the configuration of the custom-designed *in situ* DBD/FTIR reactor. The catalyst was initially reduced by H_2_/Ar (H_2_/Ar = 1:9) at 400 °C for 4 h before being pressed into a thin wafer (5 mm × 5 mm, thickness ∼ 0.5 mm). The wafer was then placed into a sample supporter. The sample supporter was placed into a flow cell (which forms a DBD reactor by adding two electrodes) that was capped at both ends by IR-transparent KBr windows. The DBD plasma can be formed between the high-voltage and ground electrodes (red and blue line, respectively) on the top and bottom of the flow cell (Figure S10). Before the *in situ* FTIR experiment, the catalyst was pretreated by plasma using 10 vol % H_2_/Ar for 10 min at 20 W, followed by sweeping with Ar for 10 min with a flow rate of 40 mL min^–1^. Then, the adsorption of CO_2_ (40 mL min^–1^) over the reduced catalyst was run at room temperature for 30 min, followed by flushing CO_2_ with H_2_ (40 mL min^–1^) for 10 min. After that, the plasma-catalytic reaction was performed in H_2_ at 20 W in a closed system (without any gas in and out). The temperature of the catalyst wafer was ∼60 °C; thus, the thermal effect on the plasma-catalytic CO_2_ hydrogenation on the Pd/ZnO surface was limited.

## Supporting Information Available

Experimental setup, kinetics analysis, characterization of the Pd–ZnO interface, reaction performance, electrical and spectroscopic diagnostics, *in situ* FTIR characterization of the catalyst surface under plasma conditions, carbon balance, and catalyst characterization results (PDF)

## Notes

The data supporting the findings of this study are available from the corresponding authors upon reasonable request.

## Dedication

Dedicated to Prof. Jianzhong Chen on the occasion of his 70th Birthday.
