# Supplementary material for: Plasma-Catalytic CO2 Hydrogenation over a Pd/ZnO Catalyst: In Situ Probing of Gas-Phase and Surface Reactions
Source: JACS Au. 2022 May 31;2(8):1800–10. doi: 10.1021/jacsau.2c00028 (PMC9400056; doi:10.1021/jacsau.2c00028)
Supplement: Supplementary file 1 — au2c00028_si_001.pdf [file au2c00028_si_001.pdf]

Supporting Information

Plasma-catalytic CO_2 hydrogenation over a Pd/ZnO catalyst: *In situ* probing of gas-phase and surface reactions

Yuhai Sun[a,b,f], Junliang Wu[a,e], Yaolin Wang[c], Jingjing Li[a], Ni Wang[c], Jonathan Harding[c], Shengpeng Mo[a], Limin Chen[a,e], Peirong Chen[a,e], Mingli Fu[a,e], Daiqi Ye*[a,e], Jun Huang*[d], Xin Tu*[c]

[a] Dr. Y. Sun, Prof. J. Wu, J. Li, Dr. S. Mo, Prof. L. Chen, Prof. P. Chen, Prof. M. Fu, Prof. D. Ye*

Guangdong Provincial Key Laboratory of Atmospheric Environment and Pollution Control, School of Environment and Energy, South China University of Technology, Guangzhou 510006, China

E-mail: cedqye@scut.edu.cn

[b] Dr. Y. Sun

School of Environmental Science and Engineering, Zhejiang Gongshang University, Hangzhou 310018, China

[c] Dr. Y. Wang, Dr. N. Wang, Jonathan Harding, Prof. X. Tu*

Department of Electrical Engineering and Electronics, University of Liverpool, Liverpool L69 3GJ, UK

E-mail: xin.tu@liverpool.ac.uk

[d] Prof. J. Huang*

Laboratory for Catalysis Engineering, School of Chemical and Biomolecular Engineering, Sydney Nano Institute, the University of Sydney, Sydney NSW 2006, Australia

E-mail: jun.huang@sydney.edu.au

[e] Prof. J. Wu, Prof. L. Chen, Prof. P. Chen, Prof. M. Fu, Prof. D. Ye*

National Engineering Laboratory for VOCs Pollution Control Technology and Equipment, South China University of Technology, Guangzhou 510006, China

[f] Dr. Y. Sun

International Science and Technology Cooperation Platform for Low-Carbon Recycling of Waste and Green Development, Zhejiang Gongshang University, Hangzhou, 310012, China

KEYWORDS: Plasma catalysis • CO_2 hydrogenation • In situ FTIR • Surface reactions • Reaction pathways

Table of Contents

1. Experimental setup

2. Kinetic analysis

3. Characterization of the Pd-ZnO interface

4. Reaction performance of CO_2 hydrogenation

5. Electrical and spectroscopic diagnostics

6. *In situ* FTIR characterization of the catalyst surface under plasma conditions

7. Carbon balance

8. Catalyst characterization results

1. Experimental setup

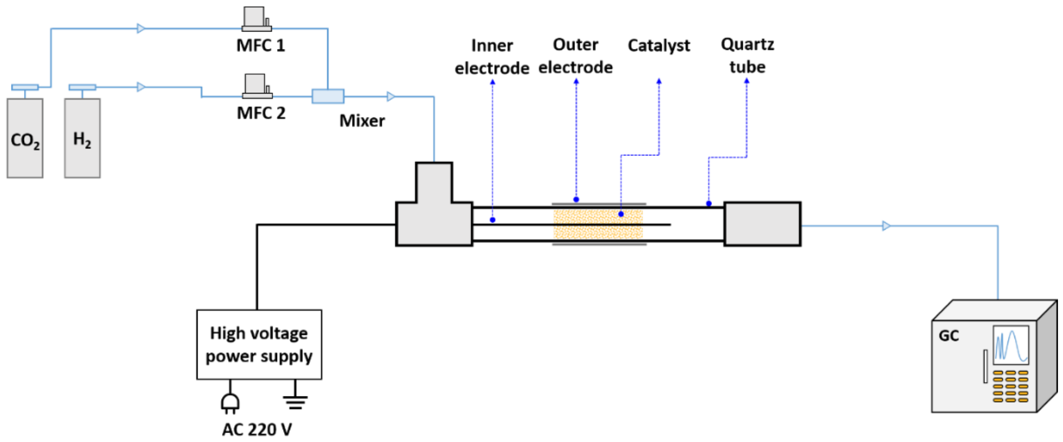

Figure S1. Schematic diagram of the experimental setup.

Definition and calculations of key reaction performances:

In the reaction, the conversion of CO_2 is defined as:

$$X_{CO_2}(\%) = \frac{CO_2 \text{ converted (mol)}}{CO_2 \text{ input (mol)}} \times 100 \qquad (1)$$

The conversion of H_2 is defined as:

$$X_{H_2}(\%) = \frac{H_2 \text{ converted (mol)}}{H_2 \text{ input (mol)}} \times 100 \qquad (2)$$

The selectivity of CO and CH_4 is calculated as:

$$S_{CO}(\%) = \frac{CO \text{ produced (mol)}}{CO_2 \text{ converted (mol)}} \times 100 \qquad (3)$$

$$S_{CH_4}(\%) = \frac{CH_4 \text{ produced (mol)}}{CO_2 \text{ converted (mol)}} \times 100 \qquad (4)$$

The CO yield and CO formation rate are defined as:

$$Y_{CO}(\%) = X_{CO_2} \times S_{CO} \times 100 \tag{5}$$

$$r_{CO}(\text{mmol h}^{-1}) = \frac{F_{CO_2}\ (\text{mL min}^{-1}) \times X_{CO_2}(\%) \times S_{CO}(\%) \times 60}{22.4\ (\text{L mol}^{-1})} \tag{6}$$

The energy efficiency for CO production is calculated as:

$$EE_{CO}\left(\text{mmol kJ}^{-1}\right) = \frac{\text{CO produced mol s}^{-1}}{\text{Power (W)}} \tag{7}$$

The carbon balance is defined as:

$$\text{CB (\%)} = \frac{CH_4 \text{ produced (mol)} + \text{CO produced (mol)}}{CO_2 \text{ converted (mol)}} \times 100 \tag{8}$$

2. Kinetic analysis

To understand the effect of CO_2 and H_2 on the plasma-catalytic CO_2 reduction, kinetic analysis was carried out to provide insights into the governing rate expression for the rate of CO production. According to previous studies [1], the power-law expression for the CO production rate in the plasma-catalytic CO_2 hydrogenation can be described by Eq. 9. Therefore, the reaction orders (*a* for CO_2 and *b* for H_2) can be determined by changing the partial pressure of one reactant (CO_2 or H_2) in excess of the other reactant (H_2 or CO_2). The partial pressure of H_2 (or CO_2) supplied was reduced and replaced with Ar to keep a constant total flow rate of 120 mL min^{-1} when keeping a constant CO_2 (or H_2) fraction and pressure (discharge power: 20 W, packing material: 0.5 g). The ratio of CO_2/(Ar + H_2) was kept at 2:1 when varying p_{H2}, while the ratio of (Ar + CO_2)/H_2 was maintained at 1:5 when changing p_{CO2} (keeping the conversion of H_2 or CO_2 lower than 15%). It is known that Ar can participate in gas-phase reactions;

however, the contribution of Ar could be minimized due to the low partial pressures of the reactants in this study. This assumption was also considered in the previous study of Barboun et al. [1].

$$r_{CO} = k\, p_{CO_2}^{a} p_{H_2}^{b} \qquad (9)$$

3. Characterization of the Pd-ZnO interface

Figure S2a shows the binding energies of Zn 2p3/2 shifted from ～1021.6 eV to ～1021.0 eV after the reduction of the catalyst, demonstrating the strong metal-support interaction (SMSI) between Pd and ZnO, which can partially reduce surface Zn^{2+} species to form a ZnO_x overlayer with the generation of abundant oxygen vacancies [2, 3]. As shown in Figure S2b, three peaks at ~531, ~532 and ~533 eV can be assigned to surface lattice oxygen (α, O_{latt}), chemisorbed oxygen species on oxygen vacancies (β, O_{ads}), and hydroxyl-like groups (γ, O_{OH}), respectively [4]. Compared to the calcined Pd/ZnO catalyst, the portion of peak β increased from 10.1% to 15.3% after the reduction of the catalyst, indicating more oxygen vacancies were formed on ZnO_x of the reduced Pd/ZnO catalyst due to the SMSI between Pd and ZnO (Table S1). Moreover, the HRTEM images of the reduced Pd/ZnO confirm the formation of a ZnO_x overlayer on the catalyst surface (Figure S3).

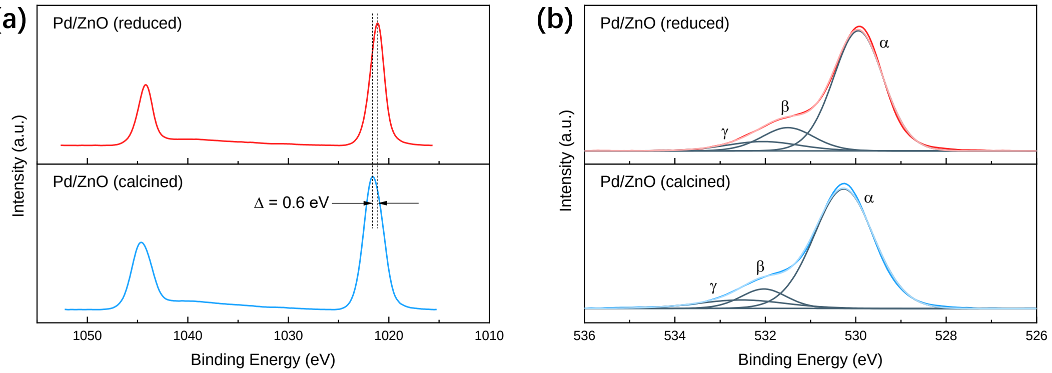

Figure S2. (a) Zn 2p and (b) O 1s XPS spectra of calcined and reduced Pd/ZnO catalysts.

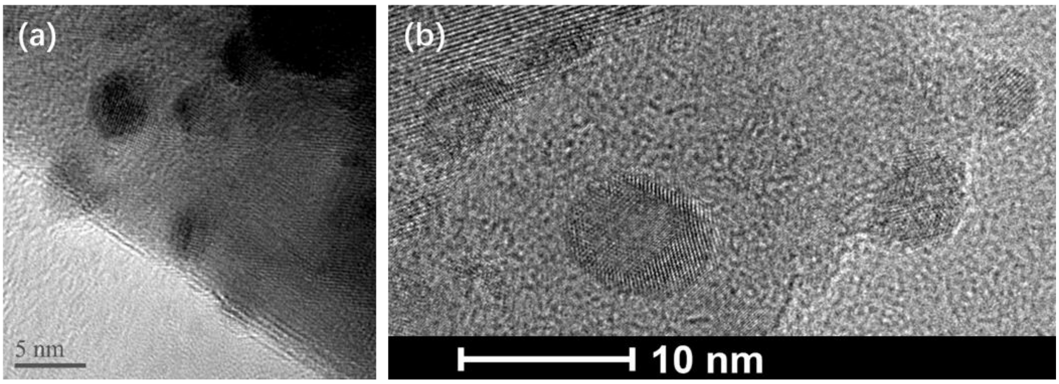

Figure S3. HRTEM images of the reduced Pd/ZnO catalyst.

4. Reaction performance of CO_2 hydrogenation

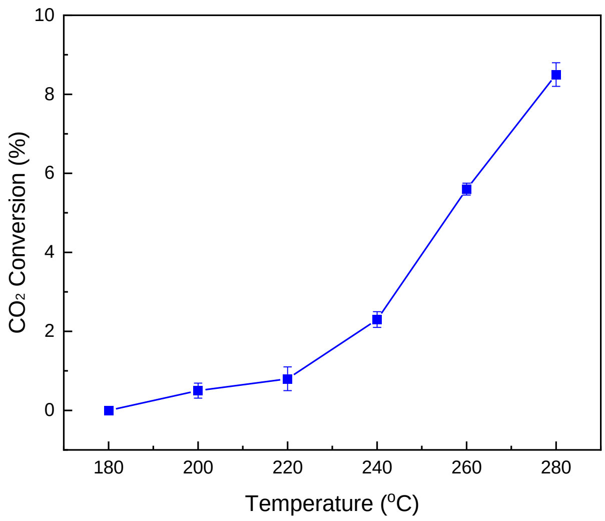

Figure S4. Effect of reaction temperature on thermal catalytic CO_2 hydrogenation over

Pd/ZnO at ambient pressure (gas hourly space velocity (GHSV) = 2200 h^{-1}, H_2/CO_2 = 3:1, total flow rate = 40 mL min^{-1}).

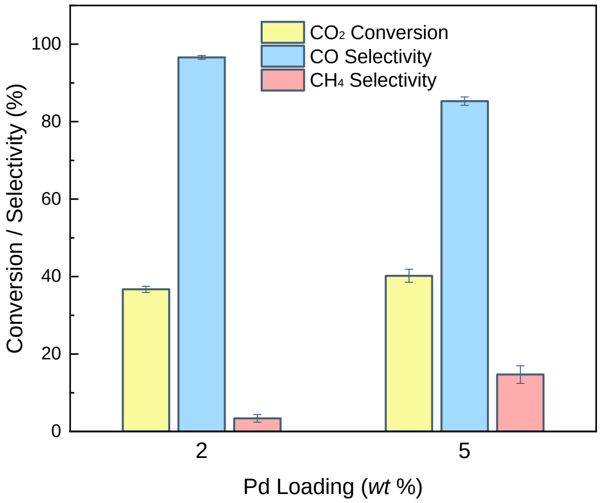

Figure S5. Effect of Pd loading on plasma-catalytic CO_2 hydrogenation (gas hourly space velocity (GHSV) = 2200 h^{-1}, H_2/CO_2 = 3:1, total flow rate = 40 mL min^{-1}, discharge power = 20 W).

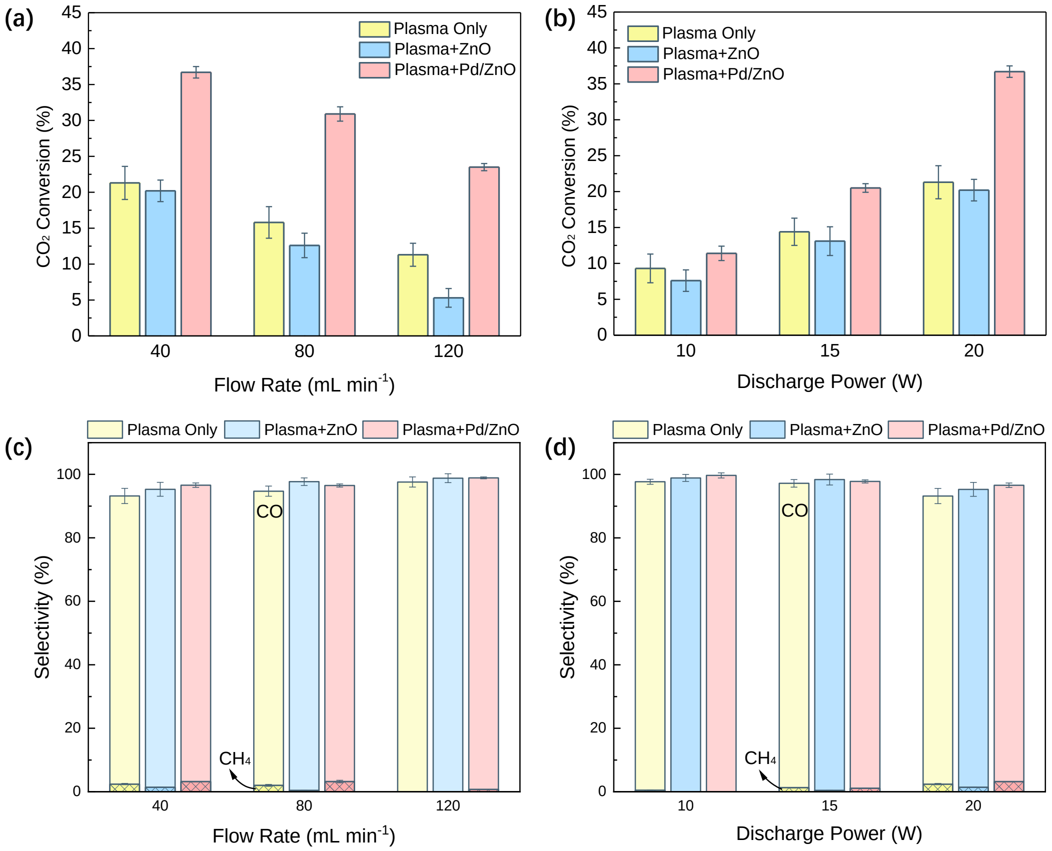

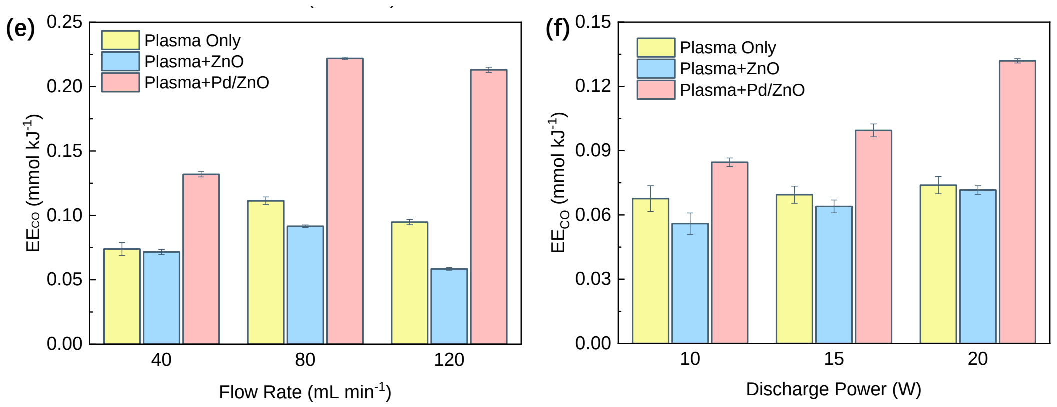

Figure S6. Effect of total flow rate (a, c) and discharge power (b, d,) on the reaction performance (CO_2 conversion and energy efficiency for CO production) in the plasma CO_2 hydrogenation with and without packing (H_2/CO_2 = 3:1; fixed discharge power = 20 W for a and c; fixed total flow rate = 40 mL min^{-1} for b and d).

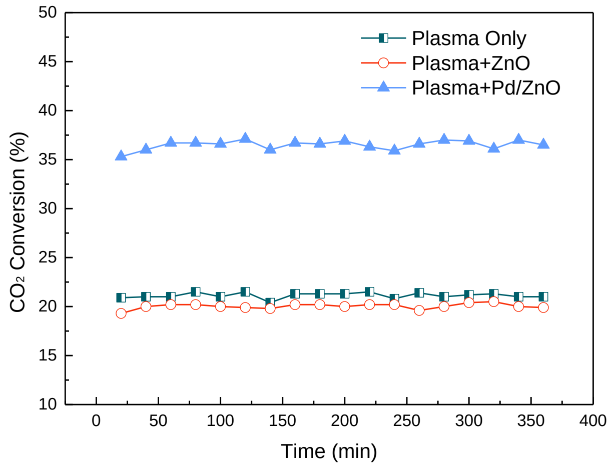

Figure S7. Stability test of CO_2 hydrogenation with and without packing (H_2/CO_2 = 3:1, total flow rate = 40 mL min^{-1}, discharge power = 20 W)

5. Electrical and spectroscopic diagnostics

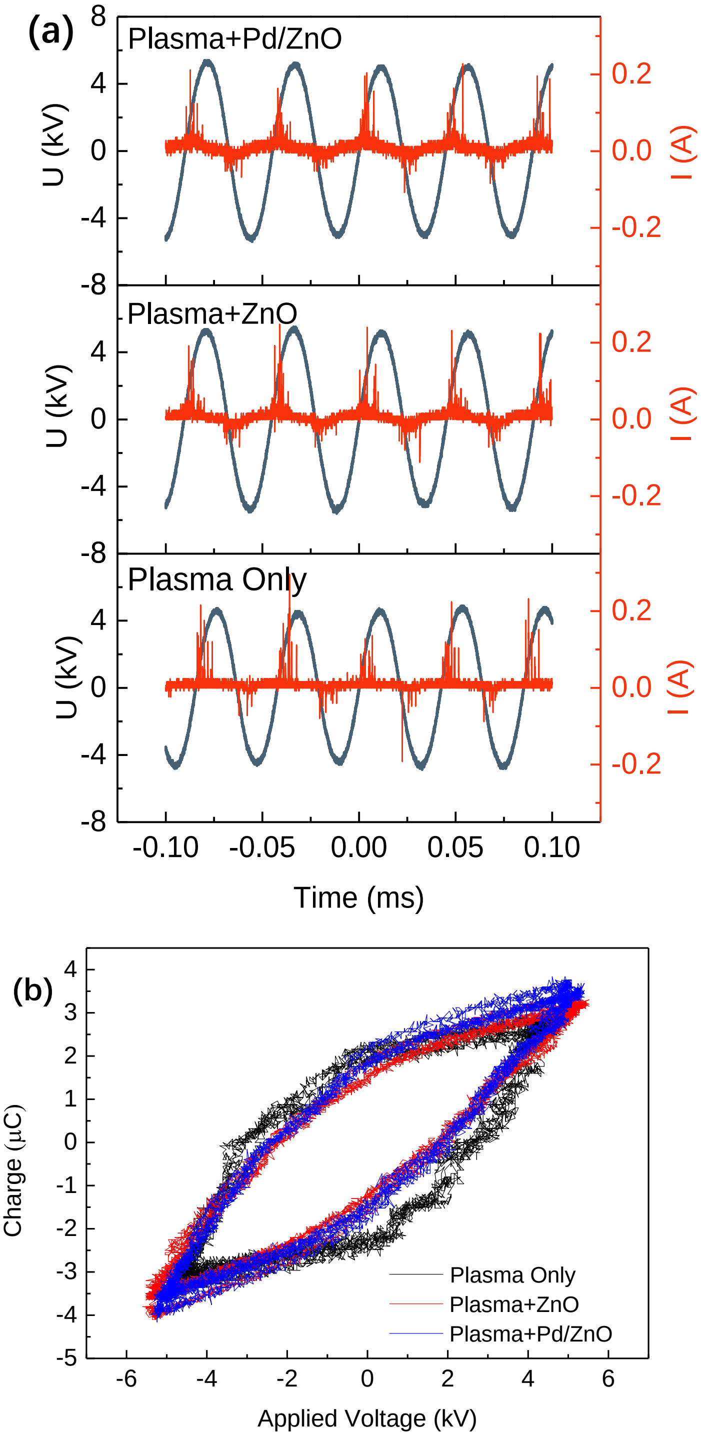

Figure S8. (a) Electrical signals; (b) Q-U Lissajous figures of the discharges. The peak-to-peak applied voltage was 9.2 kV, 11.2 kV and 11.5 kV for Plasma Only, Plasma + ZnO and Plasma + Pd/ZnO, respectively (H_2/CO_2 = 3:1, total flow rate = 40 mL min^{-1}, discharge power = 20 W).

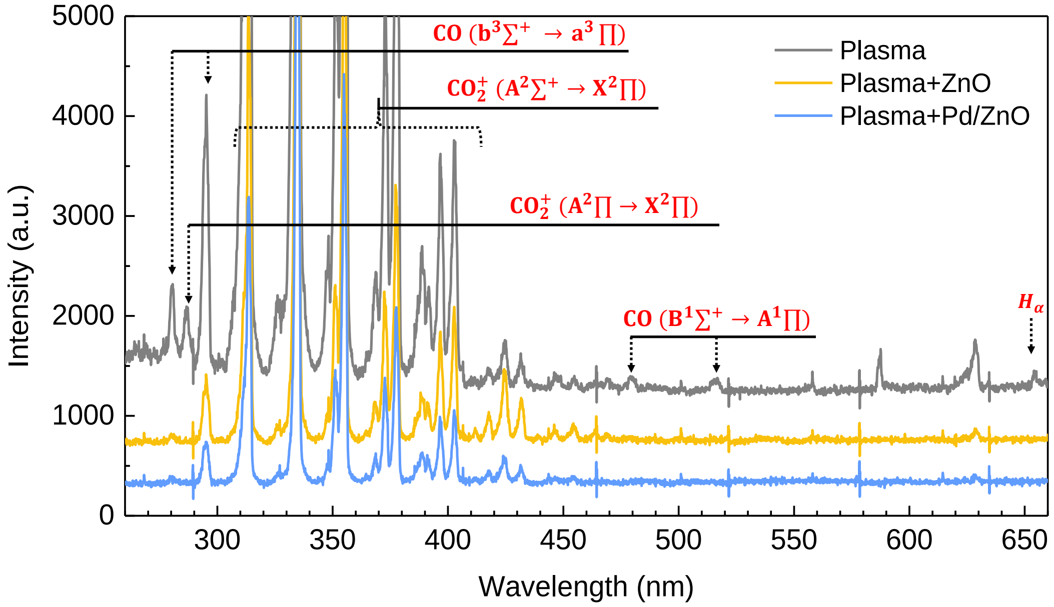

Figure S9. Emission spectra of the plasma CO_2 hydrogenation with and without packing (H_2/CO_2 = 3:1, total flow rate = 40 mL min^{-1}, discharge power = 20 W).

6. *In situ* FTIR characterization of the catalyst surface under plasma conditions

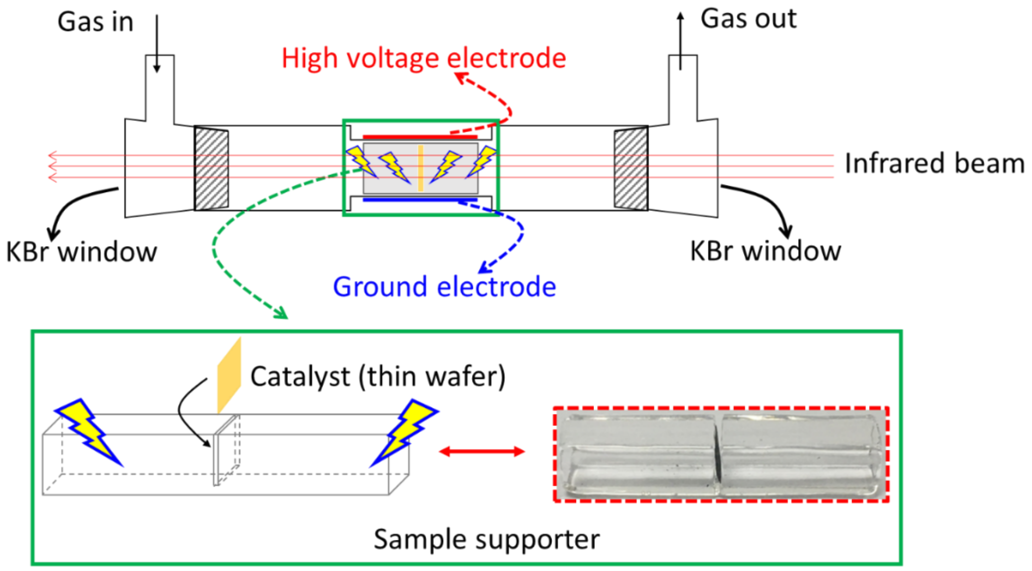

Figure S10. Scheme of the custom-designed *in situ* DBD/FTIR reactor for the analysis

of plasma-assisted surface reactions.

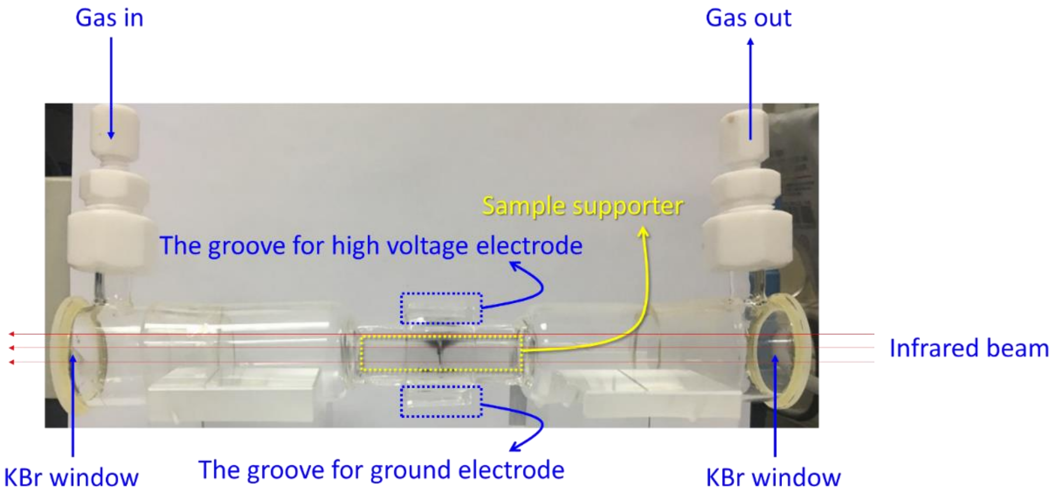

Figure S11. Photo of the *in situ* DBD/FTIR reactor for the analysis of plasma-assisted surface reactions.

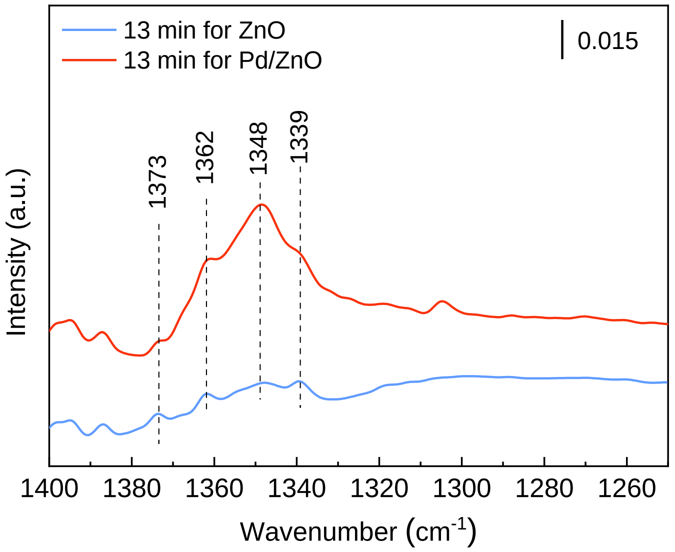

Figure S12. *In situ* FTIR spectra of ZnO and Pd/ZnO during the plasma-catalytic H_2 hydrogenation of surface adsorbed CO_2 (Corresponding to Figures 4b and 4d at 13 min).

7. Carbon balance

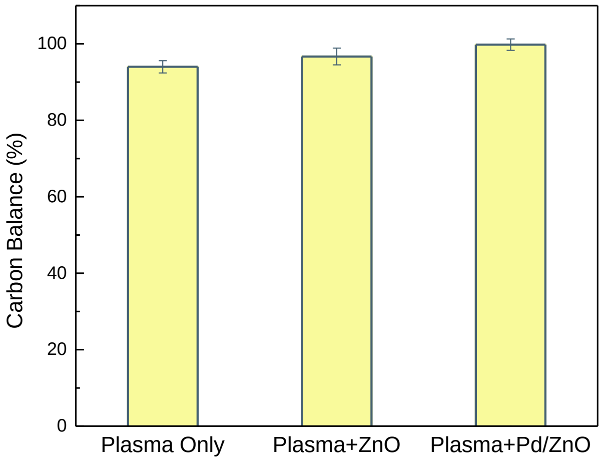

Figure S13. Carbon balance of plasma CO_2 hydrogenation with and without packing (H_2/CO_2 = 3:1, total flow rate = 40 mL min^{-1}, discharge power = 20 W)

8. Catalyst characterization results

The XRD patterns of the calcined, reduced and spent catalysts only exhibited ZnO peaks, suggesting that Pd nanoparticles (NPs) could be highly dispersed on the catalyst surface (Figures S14-S15). Figure S16 shows the XPS analysis of the surface Pd chemical state of Pd/ZnO. The calcined Pd/ZnO catalyst showed a peak of Pd^{2+} at 336.2 eV, while the XPS of the reduced and spent Pd/ZnO exhibited a peak at 335.1 eV, which can be associated with the formation of Pd NPs on the ZnO surfaces [5]. The BET specific surface area of calcined, reduced and spent Pd/ZnO catalysts was 24.9, 18.4 and 16.2 m^2/g, respectively (Figure S17 and Table S4). Compared to the calcined Pd/ZnO, the decreased specific surface area of the reduced and spent Pd/ZnO catalyst can be ascribed to the presence of Pd NPs on the catalyst surface. The catalyst

characterization showed that the properties (pore size, crystal structure, Pd surface state and morphology) of the Pd/ZnO catalyst were almost unchanged after 6 h plasma reaction (Figures S14-S19).

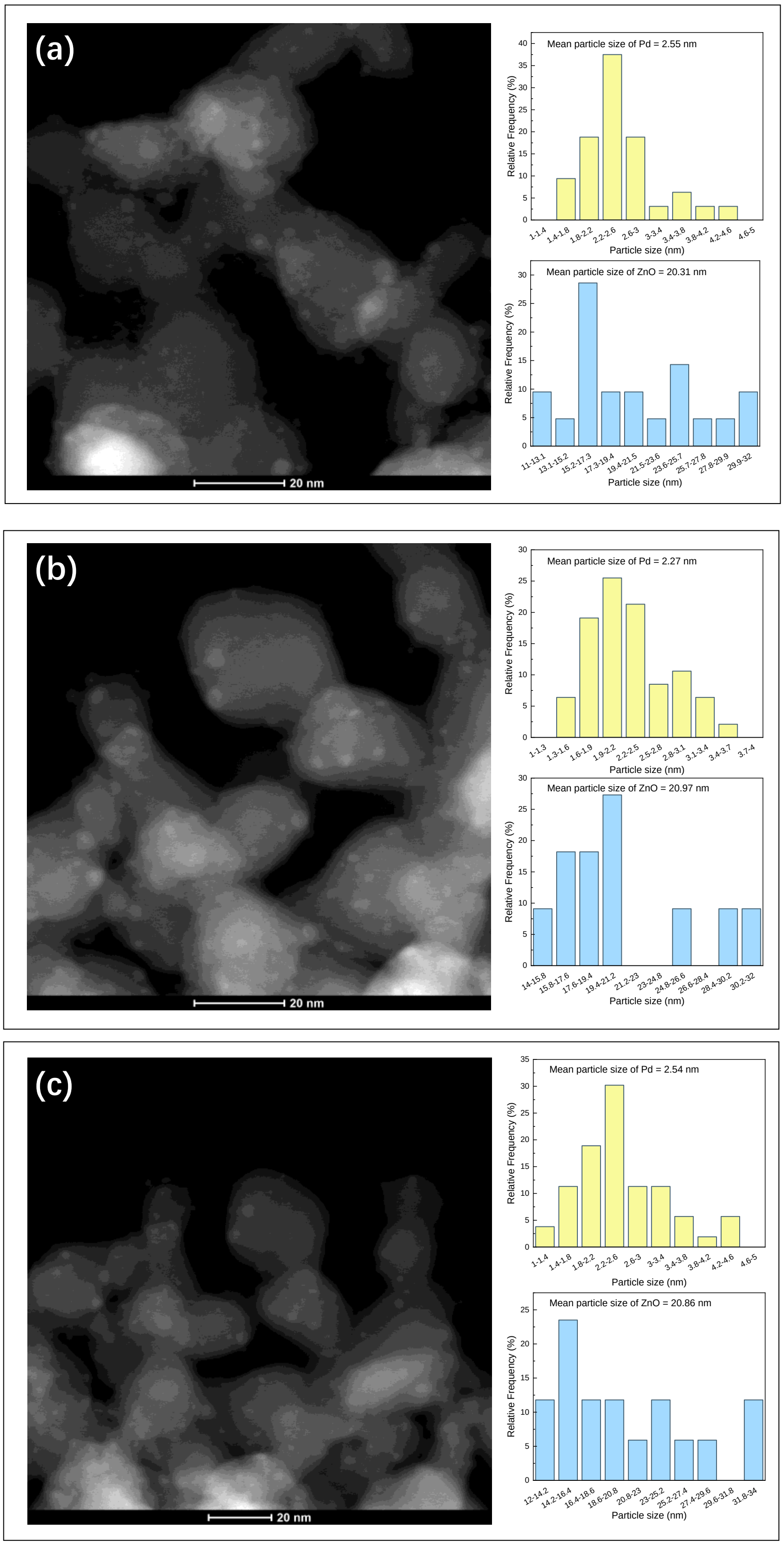

Figure S14. STEM-HAADF image of (a) calcined, (b) reduced and (c) spent Pd/ZnO.

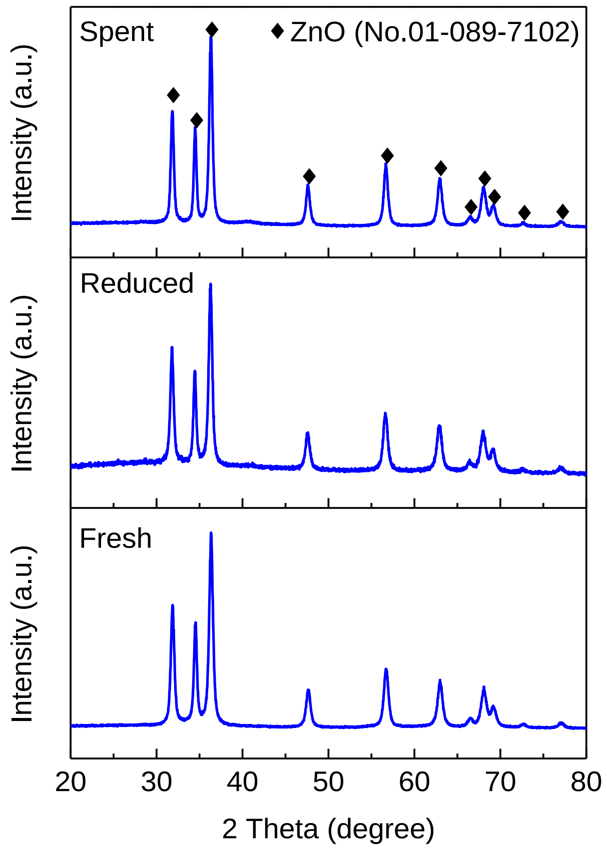

Figure S15. XRD patterns of calcined, reduced and spent Pd/ZnO catalysts.

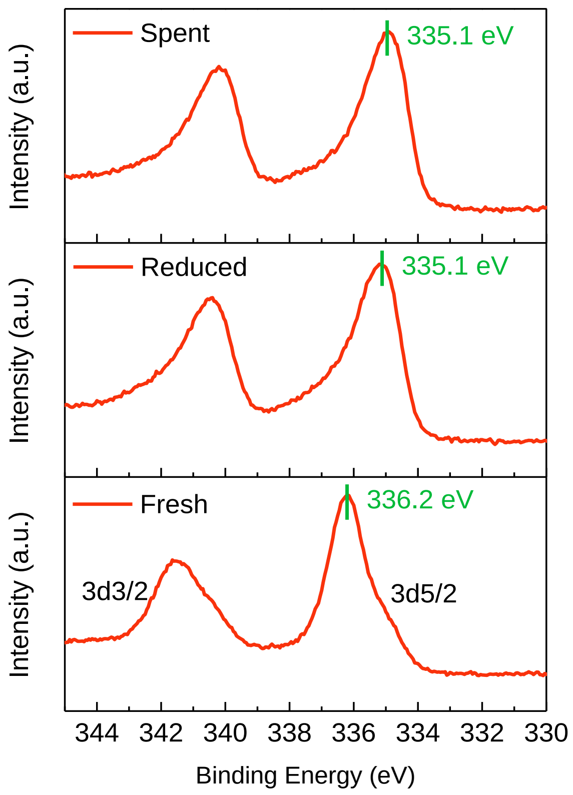

Figure S16. XPS spectra of Pd 3d for calcined, reduced and spent Pd/ZnO catalysts.

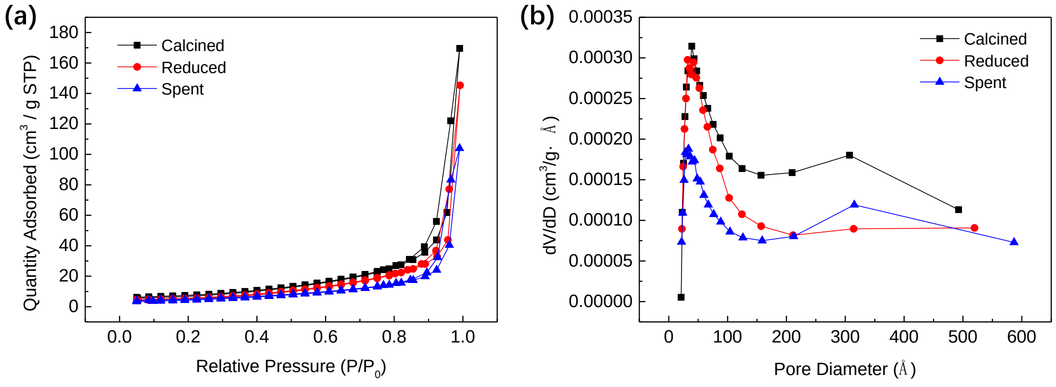

Figure S17. (a) Nitrogen adsorption-desorption isotherms and (b) the pore diameter of calcined, reduced and spent Pd/ZnO catalysts.

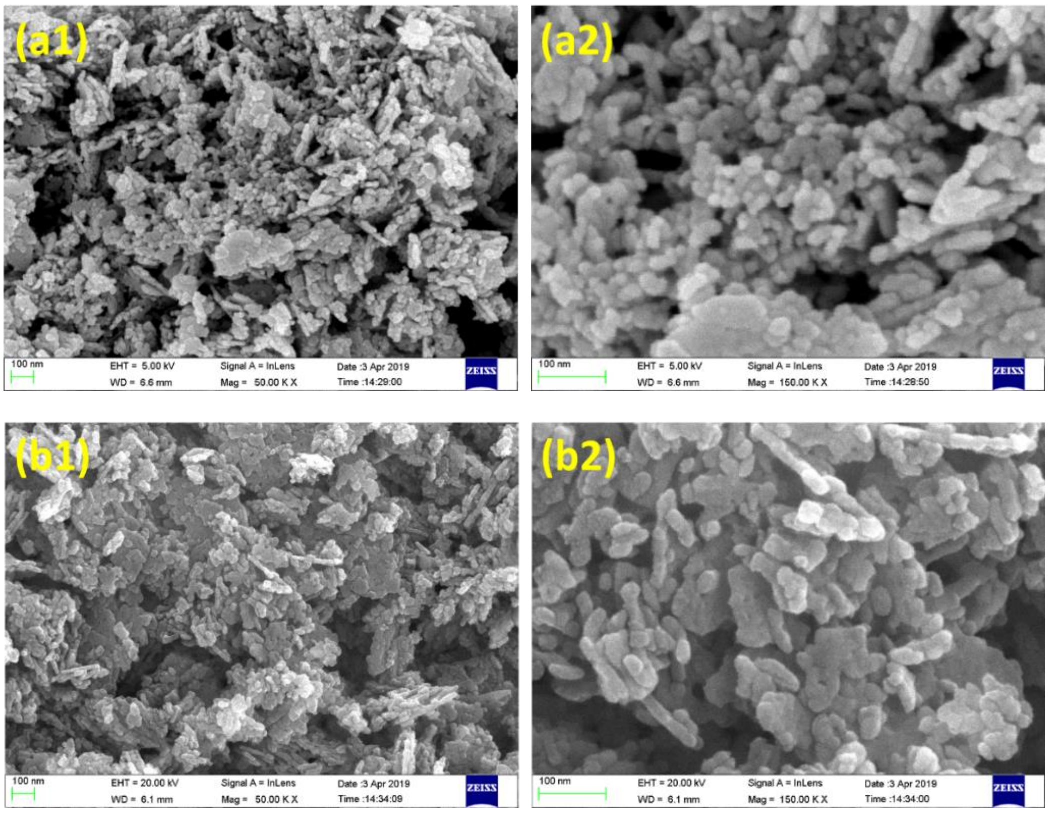

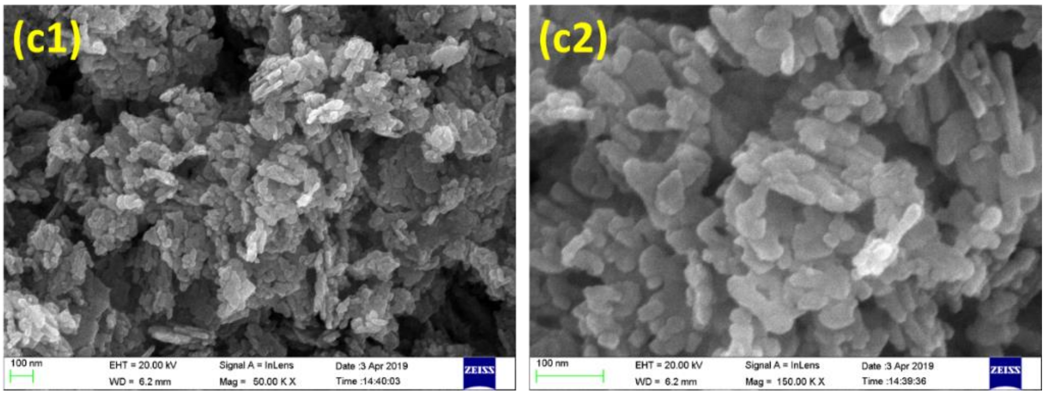

Figure S18. SEM images of (a1-a2) calcined, (b1-b2) reduced and (c1-c2) spent Pd/ZnO catalysts.

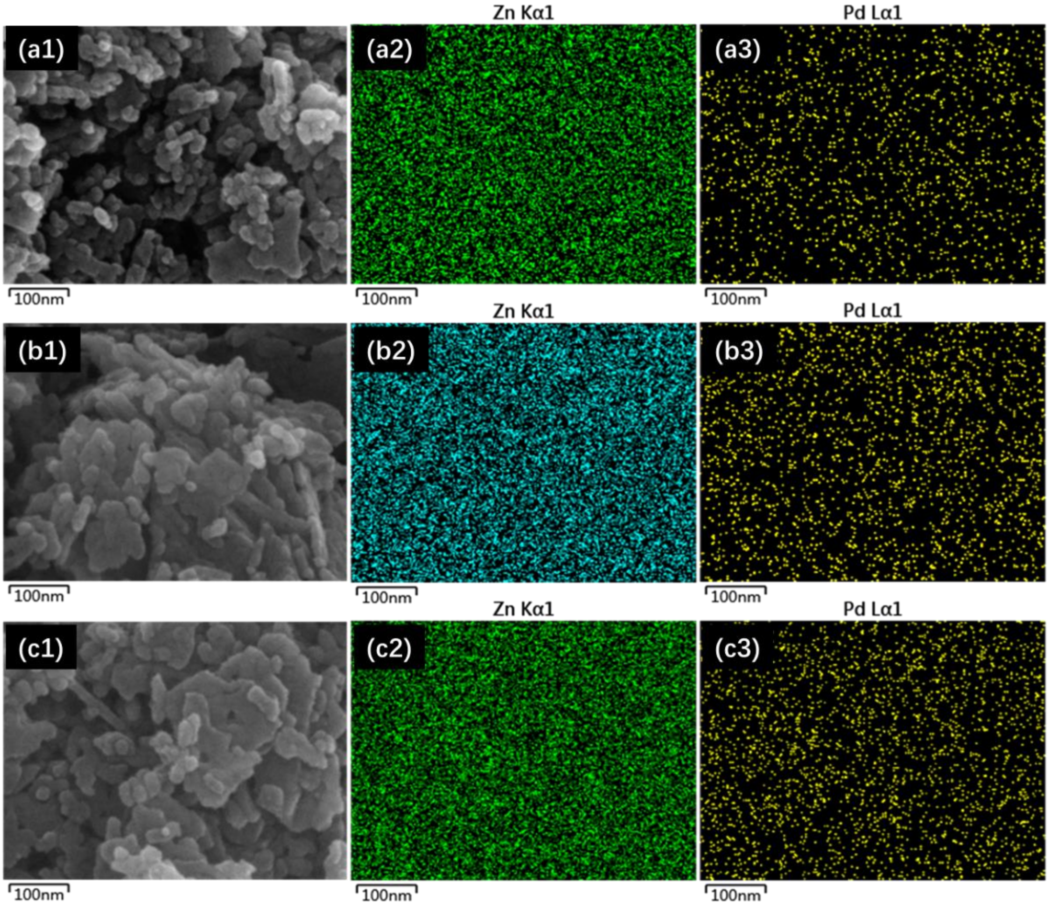

Figure S19. Elemental mapping and their corresponding SEM images of the (a1-a3) calcined, (b1-b3) reduced and (c1-c3) spent Pd/ZnO.

Table S1. XPS data of calcined (cal) and reduced (red) Pd/ZnO.

Sample	α		β		γ	
	B.E.[a] (eV)	Content (%)	B.E.[a] (eV)	Content (%)	B.E.[a] (eV)	Content (%)
Pd/ZnO (cal)	530.3	81.6	531.9	10.1	532.5	8.3
Pd/ZnO (red)	529.9	76.2	531.5	15.3	532.1	8.4

[a] Binding Energy (B.E.)

Table S2. H_2-TPD-MS analysis of ZnO and Pd/ZnO.

**Sample**	**α**		**β**		**γ**		**Total [a]**
	**T (°C)**	**I**	**T (°C)**	**I**	**T (°C)**	**I**	**I [b]**

**ZnO**	129.4	32.1	——		419.2	76.9	109.0
**Pd/ZnO**	92.4	47.2	221.7	100.0	411.0	228.7	461.2
	147.8	85.3					

[a] "Total" means the H_2 desorption amount calculated by $\alpha + \beta + \gamma$.

[b] "I" means the amount of H_2 desorption (determined by the peak area) normalized against the peak area (area intensity of 100) marked in Figure 2a.

Table S3. CO_2-TPD-MS analysis of ZnO and Pd/ZnO.

**Sample**	**α**		**β**		**γ**		**Total** [a]
	**T (°C)**	**I**	**T (°C)**	**I**	**T (°C)**	**I**	**I** [b]
**ZnO**	73.2	13.5	268.8	9.7	558.9	82.5	108.7
	139.4	3.0					
**Pd/ZnO**	86.8	37.5	256.2	100.0	458.3	6.3	220.1
	151.1	8.0	335.0	68.3			

[a] "Total" means the CO_2 desorption amount calculated by $\alpha + \beta + \gamma$.

[b] "I" means the amount of H_2 desorption (determined by the peak area) normalized against the peak area (area intensity of 100) marked in Figure 2b.

Table S4. BET analysis of the Pd/ZnO catalysts

Catalyst	BET surface area (m^2/g)	Pore volume (cm^3/g)	Pore diameter (nm)

Calcined	24.9	0.15	24.8
Reduced	18.4	0.09	26.1
Spent	16.2	0.10	26.6

References

[1] P. Barboun, P. Mehta, F.A. Herrera, D.B. Go, W.F. Schneider, J.C. Hicks, Distinguishing Plasma Contributions to Catalyst Performance in Plasma-Assisted Ammonia Synthesis, ACS Sustain. Chem. Eng., 7 (2019) 8621-8630.

[2] C. Huang, J. Wen, Y. Sun, M. Zhang, Y. Bao, Y. Zhang, L. Liang, M. Fu, J. Wu, D. Ye, L. Chen, CO_2 Hydrogenation to Methanol over Cu/ZnO Plate Model Catalyst: Effects of Reducing Gas Induced Cu Nanoparticle Morphology, Chem. Eng. J., 374 (2019) 221-230.

[3] P. Kast, M. Friedrich, F. Girgsdies, J. Kröhnert, D. Teschner, T. Lunkenbein, M. Behrens, R. Schlögl, Strong Metal-Support Interaction and Alloying in Pd/ZnO Catalysts for CO Oxidation, Catal. Today, 260 (2016) 21-31.

[4] W. Wang, Z. Qu, L. Song, Q. Fu, Probing into the Multifunctional Role of Copper Species and Reaction Pathway on Copper-Cerium-Zirconium Catalysts for CO_2 Hydrogenation to Methanol Using High Pressure in situ DRIFTS, J. Catal., 382 (2020) 129-140.

[5] E. Nowicka, S.M. Althahban, Y. Luo, R. Kriegel, G. Shaw, D.J. Morgan, Q. He, M. Watanabe, M. Armbrüster, C.J. Kiely, G.J. Hutchings, Highly Selective PdZn/ZnO Catalysts for the Methanol Steam Reforming Reaction, Catal. Sci. Technol., 8 (2018) 5848-5857.
